# Interpretable QSAR, External PubChem Validation, and Coordination-Aware Docking Enable Tiered Prioritization of Carbonic Anhydrase I Inhibitors

**DOI:** 10.3390/ph19050778

**Published:** 2026-05-15

**Authors:** Alaa M. Elsayad, Khaled A. Elsayad

**Affiliations:** 1 Biomedical Group, Department of Electrical Engineering, College of Engineering, Prince Sattam Bin Abdulaziz University, Wadi Alddawasir 11991, Saudi Arabia; 2 Pharmacy Department, Cairo University Hospitals, Cairo University, Cairo 11662, Egypt; khaled.al.elsayad@std.pharma.cu.edu.eg

**Keywords:** carbonic anhydrase I, QSAR regression, interpretable machine learning, AlvaDesc-2D descriptors, external validation, PubChem virtual screening, coordination-aware docking, AutoDock Vina, SwissADME

## Abstract

**Background/Objectives:** Carbonic anhydrase I (CAI) is a zinc-dependent metalloenzyme whose inhibitor discovery requires both effective navigation of chemical space and explicit evaluation of coordination-credible binding hypotheses. We aimed to develop an interpretable and reproducible QSAR-to-structure workflow for CAI inhibitor discovery. The workflow links potency prediction with zinc-site plausibility and early developability to support decision-oriented prioritization of new CAI inhibitor candidates. **Methods:** CAI inhibitors were retrieved from ChEMBL (CHEMBL261) and modeled as $pK_{i}=9-{log}_{10}(K_{i}\;[nM])$. AlvaDesc v3.0.8 generated 4224 2D descriptors, which were reduced using train-only preprocessing, variance filtering, correlation pruning, and bagged-tree ranking to a top-100 panel. Five regressors (elastic net, CART, bagging, GB, and XGB) were benchmarked on a held-out test set. Potent ChEMBL seeds (*K_i_* ≤ 10 nM) were used for a 90% 2D similarity PubChem expansion. Predicted hits were then externally validated using independently available PubChem CAI $K_{i}$ records. Ten novel candidates lacking CAI $K_{i}$ data were docked to CAI (PDB: 1AZM) via SwissDock AutoDock Vina in neutral and relevant anionic states, with pose selection constrained by a Zn-donor filter (Zn-N/O ≤2.6 Å). SwissADME was used to profile physicochemical space, alerts, and absorption/distribution proxies. **Results:** The bagging model showed the best test generalization ($R^{2}=0.646$; RMSE = 0.61; MAE = 0.45). PFI and SHAP converged on sulfur/heteroatom connectivity and polar–lipophilic organization as dominant potency drivers. PubChem expansion yielded 25,315 analogs and 233 candidates at predicted $pK_{i}\geq 8.0$; external validation on 145 CAI-measured hits gave $R^{2}=0.358$ (RMSE = 0.456; MAE = 0.320). Across 20 ligand/protomer docking runs, 12 produced canonical Zn-anchored poses (10 Zn-N; 2 Zn-O). SwissADME indicated consensus logP values from −0.65 to 3.21, 0/10 PAINS alerts, and predominantly favorable drug-likeness (8/10 with zero Lipinski violations), supporting tiered advancement. **Conclusions:** Integrating interpretable QSAR, external PubChem validation, coordination-aware docking, and SwissADME yields a practical triage framework for CAI inhibitor discovery. The resulting tiered shortlist identifies two Zn-N-anchored N-alkyl sulfamides (CIDs 103935964 and 112684680) and one Zn-O-anchored carboxylate control (CID 122367674) as highest-priority computational hypotheses for staged biochemical evaluation.

## 1. Introduction

Carbonic anhydrases (CAs) are a ubiquitous family of zinc-dependent metalloenzymes that catalyze the reversible hydration of carbon dioxide, thereby supporting acid–base regulation, electrolyte balance, CO_2_ transport, and metabolic buffering across mammalian tissues [1]. Among the human α-CA isoforms, carbonic anhydrase I (CAI) is highly abundant in erythrocytes and is also expressed in multiple peripheral tissues. Beyond its canonical intracellular role, accumulating evidence links altered CAI expression/activity—and, in some contexts, extracellular release—to hematologic, vascular, inflammatory, and metabolic disturbances, elevating CAI from a “housekeeping” enzyme to a biologically and pharmacologically relevant target [2]. While CA inhibition remains clinically established in indications such as glaucoma and diuresis, expanding interest in CA modulation across ophthalmic, vascular, inflammatory, metabolic, and other systemic settings has intensified the need for inhibitors that combine high potency with improved isoform-level selectivity and acceptable developability profiles [3].

The medicinal chemistry of CA inhibition is dominated by ligands bearing zinc-binding groups (ZBGs), most notably primary sulfonamides (–SO_2_NH_2_) and closely related sulfamides/sulfamates, which inhibit catalysis by displacing the zinc-bound water/hydroxide and coordinating to the catalytic Zn^2+^ [4,5]. However, inhibitory potency is strongly influenced by protonation state, substitution pattern, and overall scaffold context. This challenge is particularly important for CAI because the active-site architecture is highly conserved across human CA isoforms, making selective optimization difficult and increasing the likelihood of unwanted cross-isoform inhibition. Accordingly, CAI inhibitor discovery requires strategies that move beyond potency ranking alone and instead reveal chemically interpretable structure–activity patterns that can guide rational prioritization within a closely related enzyme family.

Quantitative structure–activity relationship (QSAR) modeling provides a scalable route for connecting chemical structure to experimentally measured inhibitory potency and for prioritizing candidates before synthesis or biochemical evaluation. In particular, 2D descriptor-based QSAR offers a practical balance between computational efficiency and interpretability on large, structurally heterogeneous datasets. Descriptor engines such as AlvaDesc encode molecular constitution, topology, fragment composition, electronic proxies, and surface-related features through thousands of chemically meaningful numerical descriptors [6]. Yet the predictive value of such high-dimensional representations depends critically on rigorous curation, leakage-controlled preprocessing, and disciplined feature reduction, because collinearity and inadvertent information leakage can inflate apparent performance and undermine deployability [6,7]. For this reason, robust QSAR workflows should (i) fit all transformations on training data only, (ii) apply the learned transforms unchanged to test/external sets, and (iii) accompany performance reporting with explainability analyses that clarify which chemical themes drive predictions. In this context, permutation feature importance (PFI) and SHAP provide complementary views of descriptor relevance and can convert a high-performing model into an actionable medicinal chemistry tool by highlighting stable driver families and split-robust signal [8,9].

A reliable QSAR model can further serve as an engine for prospective chemical-space expansion. Public repositories such as PubChem enable high-similarity retrieval of analogs around potent seed inhibitors, allowing model-guided screening of near-neighbor compounds beyond the original training distribution [10]. However, ligand-based prioritization alone cannot ensure mechanistic plausibility—particularly for zinc metalloenzymes. Structure-based triage remains valuable for testing whether predicted hits can adopt catalytically credible binding hypotheses within the CAI active site. Docking to metalloproteins is nontrivial because generic scoring functions often under-represent metal–ligand coordination physics, yielding poses that are sterically acceptable yet chemically inconsistent with Zn^2+^ anchoring. Accordingly, docking-based evaluation of CAI candidates should be anchored to crystallographic context and supplemented with explicit zinc-geometry plausibility filters, rather than relying on docking scores alone [11]. From a translational perspective, early developability screening is essential, as high biochemical potency and structurally plausible binding modes do not guarantee successful progression to viable drug candidates. Indeed, unfavorable absorption, distribution, metabolism, and excretion (ADME) properties remain a leading cause of attrition in drug discovery, frequently emerging after substantial resources have already been invested [12,13]. Consequently, insufficient ADME characterization continues to represent a major bottleneck in the transition from hit identification to clinical development.

In this study, we present an interpretable and mechanistically grounded QSAR-to-structure workflow for CAI inhibitor discovery that integrates predictive modeling, descriptor-level explanation, prospective screening, coordination-aware docking, and developability assessment. Specifically, the workflow (i) compiles and curates CAI inhibition data as *K_i_* -derived *pK_i_* values, (ii) computes comprehensive AlvaDesc-2D descriptors and applies leakage-controlled preprocessing with variance filtering, correlation pruning, and bagged-tree feature ranking to obtain a compact descriptor panel, (iii) benchmarks multiple regression algorithms and selects the model with the best generalization on a held-out test set, (iv) performs a high-similarity PubChem expansion and evaluates transferability using independently available PubChem CAI records; (v) triages a structurally diverse subset of novel QSAR-prioritized candidates via coordination-aware docking against CAI with explicit Zn–donor geometry constraints; and (vi) profiles the docked shortlist using SwissADME. By coupling interpretable ligand-based learning with coordination-consistent structural triage and early ADME filtering, this integrated framework provides a reproducible route for prioritizing CAI inhibitor candidates for downstream experimental validation.

### Related Work

Recent advances in carbonic anhydrase (CA) research indicate that credible inhibitor discovery should be grounded in three closely linked considerations: isoform-specific physiology and disease context, the selectivity and polypharmacology constraints inherent to the CA family, and computational workflows that remain chemically faithful to zinc coordination when moving from ligand-based prioritization to structure-based triage. Broad pharmacological syntheses further show that CA inhibitors—historically regarded as “mature” drugs—continue to reveal new therapeutic opportunities as well as new liabilities across unexpected indications, reinforcing the need for careful isoform-aware profiling rather than potency-only optimization [14]. In parallel, the emergence of dual-target CA inhibitor design further highlights the value of integrated workflows capable of interpreting structure–activity patterns and supporting rational prioritization across chemically diverse scaffolds [15]. These issues are especially relevant to CAI, where strong active-site conservation across isoforms increases the risk of cross-reactivity and where biological context may influence the translational significance of potency gains.

CAI physiology and vascular relevance: CAI has long been viewed primarily as an abundant erythrocytic isozyme responsible for intracellular CO_2_ handling. However, emerging evidence suggests that its physiological role may be broader under stress conditions. Doroudian and Gailer demonstrated that CAI released upon erythrocyte rupture remains structurally intact and freely diffusing in plasma, rather than being rapidly degraded or sequestered [2]. This observation is mechanistically important because it supports the view that CAI may remain extracellularly available in hemolytic contexts. It therefore provides a plausible link between hemolysis-associated enzyme release and downstream biochemical effects in blood-associated microenvironments, including the blood-endothelium interface. Vascular-focused analyses further place CA activity within the broader context of systemic hemodynamic regulation. García-Llorca et al. reviewed evidence indicating that several carbonic anhydrase isoforms, including CAI, contribute to the regulation of vascular tone and regional blood flow, and that pharmacological inhibition of these isoforms may elicit vasodilatory responses in ocular and cerebral vascular beds [16]. Together, these findings reinforce that CA inhibition can produce system-level physiological effects, highlighting why CAI inhibitor development should consider exposure, tissue distribution, and isoform cross-reactivity—especially for chemotypes expected to circulate systemically.

Therapeutic landscape and the selectivity–polypharmacology problem: CA inhibitors remain foundational in ophthalmology, yet modern reviews increasingly position CA modulation as a platform with expanding therapeutic scope. A scoping review of systemic CA inhibitors in common ophthalmic diseases confirms sustained clinical utility while emphasizing that systemic adverse effects remain central to real-world prescribing and risk-benefit considerations [4]. More broadly, Giovannuzzi and Supuran synthesize the evolving landscape of CA modulators, highlighting emerging strategies while reiterating that isoform selectivity remains a central challenge because catalytic-site architectures are highly conserved across the CA family [17]. In this context, potency alone is not a sufficient optimization objective. Selectivity is inseparable from polypharmacology, and cross-isoform inhibition may be beneficial in some settings but can also generate clinically relevant liabilities when systemic exposure is required, particularly when off-target inhibition of ubiquitous isoforms such as CA II contributes to adverse effects [18]. Complementing this view, broader CAI overviews show that “old” CA chemotypes continue to find new applications, reinforcing the need for discovery strategies that balance potency with isoform selectivity, exposure context, and downstream pharmacology. Finally, the increasing interest in dual-target CA inhibitors further raises the bar for mechanistically interpretable workflows that can rationalize potency drivers while simultaneously tracking selectivity-related liabilities.

Computational discovery pipelines: from similarity expansion to coordination-aware docking: Structure-based virtual screening and docking remain central to CA inhibitor discovery, particularly when seeking to expand beyond classical sulfonamide chemotypes. Cheng and colleagues reported a structure-based screening workflow for CA IX that illustrates a generalizable principle: docking hypotheses are most credible when coupled to orthogonal validation layers and when exploration extends beyond canonical zinc-binding paradigms [19]. Importantly, this CA IX study is cited here as a methodological exemplar rather than CAI-specific evidence, because isoform differences can alter binding determinants and selectivity logic. In parallel, QSAR remains a core component of ligand-based modeling for CA inhibition when designed to balance performance with interpretability. Hammoudan and Chafi provide an example of descriptor-driven QSAR for CA inhibitor series and emphasize validation practices aligned with contemporary best-practice expectations [20]. Multi-modal workflows that integrate QSAR with docking and property filters are also increasingly common; for example, Naouri and co-workers combined 3D-QSAR, docking, and ADMET analysis for CA XII inhibitors to derive statistically robust models and interaction hypotheses [21]. As with CA IX, CA XII is cited here to illustrate an integrated computational pattern rather than to support CAI-specific conclusions. A persistent limitation across such pipelines is that generic docking scoring functions often under-represent metal-ligand coordination energetics, yielding poses in zinc metalloenzymes that appear sterically plausible yet lack chemical consistency with catalytic-site coordination. AutoDock Vina 1.2.x introduced methodological improvements and expanded scoring options, yet zinc coordination remains a regime where pose credibility should be evaluated explicitly rather than inferred from score alone [22]. For CA targets, docking is therefore commonly anchored to high-confidence crystallographic contexts—such as CAI structures including PDB: 1AZM—and strengthened by coordination-aware post-filters (e.g., Zn–donor distance constraints) to exclude zinc-incompatible poses [23]. This shift converts docking from a score-centric ranking step into a mechanistic plausibility filter that is especially useful when triaging QSAR-prioritized candidates.

Interpretable ML and XAI for QSAR: feature attribution as a design tool: As QSAR pipelines increasingly rely on ensemble learners trained on high-dimensional descriptor spaces, explainable AI (XAI) has shifted from optional to operationally necessary—supporting mechanistic credibility, governance, and transparent model-guided decision-making. A recent survey of XAI in drug discovery emphasizes SHAP, permutation-based importance, LIME, and related attribution methods as practical tools for improving interpretability and trust in model-guided prioritization [24]. For feature selection specifically, permutation feature importance (PFI) is attractive because it is model-agnostic and directly estimates reliance on each variable by quantifying performance degradation under controlled feature shuffling [25]. Comparative evaluations of QSAR feature-selection methods further support the practical value of SHAP- and permutation-based rankings for generating stable, interpretable descriptor panels suitable for medicinal chemistry triage—particularly under unavoidable feature collinearity, where interpretation is most defensible at the descriptor-family level rather than as unique causal attribution to a single variable [26].

Summary and positioning of the present study: Collectively, recent physiology and vascular evidence positions CAI as a biologically meaningful isoform whose extracellular behavior may be relevant under hemolytic or vascular stress [2,16], while contemporary pharmacological syntheses underscore that CA inhibitor development must explicitly address isoform selectivity and polypharmacology within a highly conserved enzyme family [18]. Methodologically, CA inhibitor discovery increasingly adopts hybrid pipelines that combine QSAR-driven prioritization, similarity-based chemical-space expansion, and docking, yet also recognizes that zinc-metalloenzyme docking requires coordination-credible pose evaluation rather than reliance on docking scores alone [19,22,23]. Against this backdrop, the present work contributes an interpretable, leakage-controlled QSAR framework for CAI inhibitor discovery that is coupled to coordination-aware structural triage and early developability assessment. The study addresses a specific methodological gap: not the absence of CA inhibitor modeling in general, but the lack of a quantitative, interpretable, CAI-specific regression framework that can rank inhibitory potency on a continuous $pK_{i}$ scale, demonstrate transferability beyond internal splits through independently sourced external validation, and connect ligand-based prioritization to structurally credible zinc-site hypotheses and early developability filtering. To address this gap, we integrated QSAR regression, descriptor-level interpretability analysis, external PubChem transferability testing, zinc-geometry-constrained docking triage, and SwissADME-supported prioritization into a coherent and reproducible workflow for CAI inhibitor discovery.

## 2. Results

### 2.1. Dataset Characteristics and Descriptor Reduction

The curated CAI dataset provided a broad and information-rich activity landscape for regression modeling. After standardization, deduplication, and exclusion of compounds outside the predefined modeling range, the final ChEMBL-derived dataset comprised 6958 unique CAI inhibitors described initially by 4224 AlvaDesc 2D descriptors. Sequential train-only preprocessing substantially compressed this feature space while preserving chemically relevant information: low-variance filtering reduced the descriptor matrix to 2548 variables, correlation pruning further reduced it to 1373 descriptors, and bagged-tree ranking yielded a final top-100 descriptor panel for downstream model development. The top-100 descriptors were selected to balance predictive signal retention, interpretability, stability, and computational efficiency. This progressive reduction was important not only for computational tractability, but also for statistical stability, because it transformed a highly collinear high-dimensional representation into a more compact and interpretable descriptor space suitable for supervised learning.

**Figure 1:**
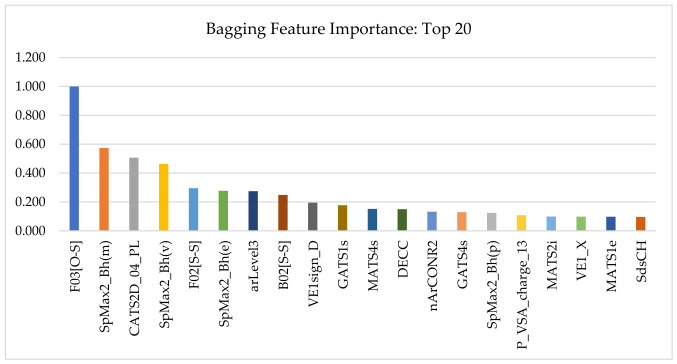
Scaled importance ranking of the top 20 AlvaDesc 2D descriptors in the final bagging ensemble model for carbonic anhydrase I (CAI) $pK_{i}$ prediction. Descriptor importances were normalized to the highest-ranked feature, F03[O–S], which was set to 1.0. The model was trained using 500 trees with a maximum depth of 50, and the displayed values represent relative feature importance within the fitted ensemble.

Importantly, the reduction process did not collapse the signal into an arbitrary or purely abstract feature subset. Rather, the highest-ranking descriptors retained in the reduced space, summarized in Table 1 and Figure 1, indicate that the surviving predictive information was concentrated in chemically meaningful descriptor families related to sulfur/heteroatom environments, global scaffold topology and size, pharmacophore-like polar–lipophilic organization, and charge/surface partitioning. The dominance of descriptors such as F03[O–S], together with the strong presence of Burden eigenvalue, CATS2D, and autocorrelation-based descriptors, suggests that feature reduction preserved the major structural patterns most relevant to CAI inhibitory potency rather than merely optimizing dimensionality numerically. In this sense, descriptor reduction served a dual role: it improved model tractability while also exposing a more interpretable representation of the CAI structure–activity landscape.

Thus, the final descriptor panel can be viewed as a condensed but chemically informative encoding of CAI-relevant SAR. Instead of distributing predictive signal diffusely across thousands of partially redundant variables, the workflow concentrated that signal into a smaller subset that already foreshadowed the main medicinal chemistry themes later confirmed by model interpretation, namely the importance of sulfonyl-proximal motifs, overall scaffold architecture, and balanced polar–lipophilic organization. The detailed implications of these ranked descriptors are discussed further in Section 2.3.

### 2.2. Comparative Regression Performance and Model Selection

**Figure 2:**
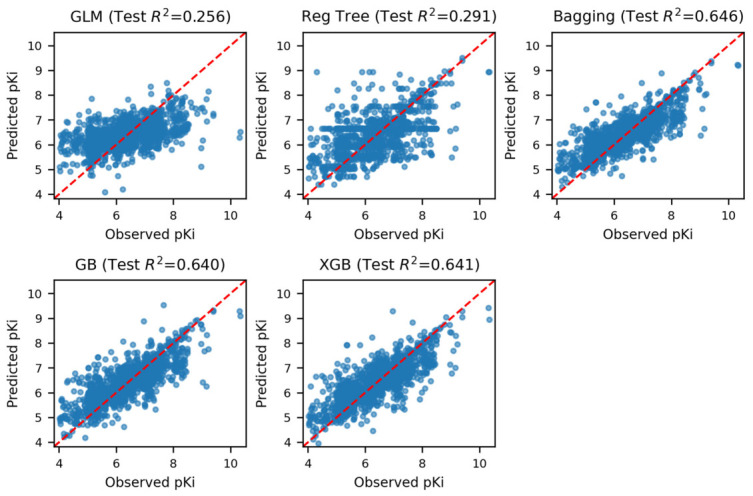
Observed versus predicted CAI inhibitory potency $\left(pK_{i}\right)$ on the held-out test set for the five regression models developed using the optimized AlvaDesc 2D descriptor panel. Panels correspond to Elastic Net (GLM), regression tree (CART), bagging, gradient boosting (GB), and XGBoost (XGB). The red dashed line denotes the identity line (Predicted = Observed). Test-set $R^{2}$ values are reported in each panel, illustrating the markedly improved generalization of the ensemble models relative to the linear and single-tree baselines, with bagging providing the best overall balance between predictive accuracy and stability.

The comparative evaluation of the five regression models revealed a clear performance hierarchy on the held-out test set (Table 2; Figure 2). The $R^{2}$, RMSE, and MAE values reported here are point estimates derived from the predefined held-out test subset. Ensemble-based learners substantially outperformed both the linear baseline and the single-tree model, indicating that CAI inhibitory potency is governed by nonlinear relationships that are not adequately captured by simple additive models or shallow partitioning alone. In particular, the strong improvement of the ensemble methods over Elastic Net and CART suggests that predictive signal in the optimized AlvaDesc 2D descriptor space arises from higher-order interactions among topological, fragment-based, and electrostatic descriptor families rather than from isolated linear contributions.

Although gradient boosting and XGBoost achieved the strongest apparent fits on the training data, this advantage did not translate into superior performance on the held-out test set. XGBoost produced the highest training accuracy $\left(R^{2}=0.98,RMSE=0.14,MAE=0.08\right)$, followed closely by gradient boosting $\left(R^{2}=0.96,RMSE=0.21,MAE=0.15\right)$. However, both models exhibited a larger train-test performance gap than the bagging ensemble. In the present descriptor setting, this pattern is consistent with partial overfitting to training-specific structure, likely reflecting the greater flexibility of boosting-based learners in a reduced yet still high-dimensional feature space. By contrast, the bagging ensemble achieved the strongest held-out generalization, with $R^{2}=0.65$, RMSE = 0.61, and MAE = 0.45, marginally outperforming gradient boosting and XGBoost on test-set $R^{2}$ while maintaining a more stable error profile. Table 2 therefore indicates that the final model was selected not on the basis of maximal in-sample fit, but on the basis of the most reliable predictive behavior under unseen data conditions.

This distinction is also evident in Figure 2, where the observed-versus-predicted plots provide a visual summary of model behavior on the held-out test set. The Elastic Net and regression tree models show broader dispersion around the identity line, consistent with underfitting and weaker predictive resolution. In contrast, the ensemble models produce visibly tighter clustering around the identity line, confirming that they recover a substantially larger fraction of the continuous $pK_{i}$ signal. Among them, the bagging model shows the most balanced pattern, with strong alignment to observed values across much of the potency range and less evidence of the exaggerated training-fit behavior observed for the more aggressive boosting models.

From a practical QSAR perspective, this result is especially important because the intended use of the model is prospective screening under moderate chemical-space variability, rather than retrospective explanation of the training set alone. Under that deployment scenario, the most useful model is not the one with the highest in-sample fit, but the one that best preserves predictive accuracy when applied to new compounds. The superior held-out generalization of the bagging ensemble therefore justified its selection as the final model for PubChem external validation, prospective hit prioritization, and subsequent docking and ADME-based triage.

These comparative results indicate that CAI potency can be predicted with meaningful accuracy from a reduced 2D descriptor representation, but that robust prediction depends on variance-controlled ensemble learning rather than on either strictly linear regression or more aggressively fit boosting models. This finding supports the use of bagging as the most appropriate compromise between predictive strength, stability, and downstream interpretability in the present CAI QSAR framework.

### 2.3. Descriptor Importance and Chemical Interpretation of the Learned Signal

**Figure 3:**
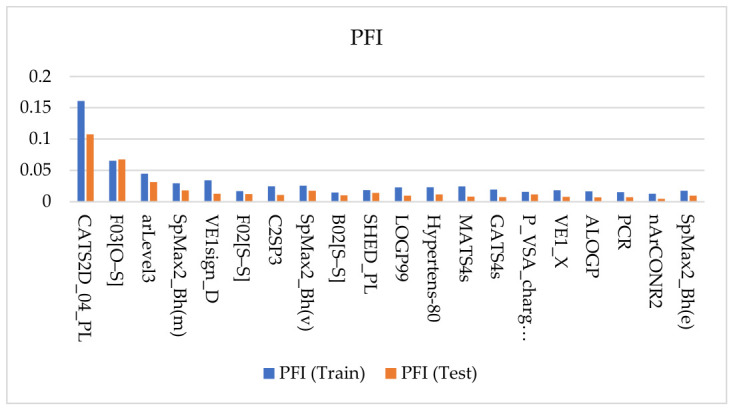
Permutation feature importance (PFI) stability across training and held-out test subsets for the final bagging QSAR model. Bars show the absolute mean PFI values of the top 20 AlvaDesc 2D descriptors. Higher values indicate greater model reliance, as measured by the reduction in predictive performance after controlled permutation of each descriptor. The paired display enables direct assessment of train-test stability in descriptor importance.

**Figure 4:**
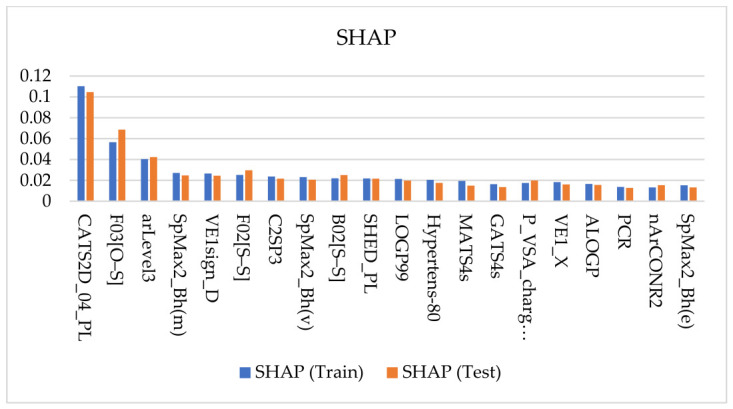
SHAP importance stability across training and held-out test subsets for the final bagging QSAR model. Bars show the absolute mean SHAP magnitude of the top 20 AlvaDesc 2D descriptors. Because SHAP values decompose predictions into additive feature contributions, the absolute mean summarizes overall descriptor influence irrespective of direction. Consistency between training and test rankings supports stable attribution and reduces concern that the learned signal is driven by split-specific artifacts.

To interrogate the descriptor-level basis of the final bagging QSAR model and to assess whether the learned structure–activity signal was stable rather than split-specific, two complementary explainability frameworks were applied: permutation feature importance (PFI) and SHAP (SHapley Additive exPlanations). For both methods, descriptor relevance was summarized as an absolute mean magnitude, such that the reported values reflect the strength of contribution to prediction irrespective of direction. The top 20 descriptors identified by each approach are shown as paired train/test summaries in Figure 3 (PFI) and Figure 4 (SHAP).

A central result of this analysis is the strong concordance between PFI and SHAP, together with the persistence of major descriptor families from the training subset to the held-out test subset. Across both explainability frameworks, the same chemically interpretable groups consistently dominated the attribution profile:

- sulfur-heteroatom connectivity and sulfonyl-associated signatures, including F03[O–S], F02[S–S], and B02[S–S];
- pharmacophore-like 2D patterning descriptors, particularly those capturing polar–lipophilic organization and interaction-center spacing, such as CATS2D_04_PL and SHED_PL;
- global topology/shape and distributed-property autocorrelation descriptors, including the SpMax2_Bh(*), GATS*, MATS*, and VE1* families.

Taken together, these patterns indicate that the model does not rely on isolated local fragments but instead integrates local chemical motifs with a broader scaffold architecture and a distributed physicochemical organization. As expected, the absolute importance magnitudes were generally lower on the test subset than on the training subset, reflecting the usual attenuation that accompanies evaluation on unseen data.

Importantly, however, the leading descriptors remained broadly consistent across partitions and across XAI methods. Features such as CATS2D_04_PL, F03[O–S], and arLevel3 retained prominent positions in both PFI and SHAP, indicating that the dominant contributors to model behavior were not artifacts of a particular split or attribution formalism. This train-test persistence strengthens confidence that the selected bagging model is capturing a reproducible structure–activity signal rather than overfitting idiosyncratic patterns in the training data.

From a chemical perspective, the XAI results converge on an interpretable view of CAI potency prediction. The prominence of F03[O–S] and related sulfur-connectivity descriptors highlights the importance of sulfonyl-adjacent environments and heteroatom-sulfur patterns, consistent with the well-established prevalence of sulfur-containing chemotypes among carbonic anhydrase inhibitors. At the same time, these descriptors should not be interpreted as proof of a specific zinc-binding mechanism in isolation; rather, they indicate that the model is highly sensitive to structural environments commonly associated with privileged CA inhibitor motifs and their substitution context.

A second major theme is the importance of polar–lipophilic organization. Descriptors such as CATS2D_04_PL and SHED_PL suggest that CAI potency is shaped not only by the presence of suitable functional groups, but also by how polar and hydrophobic elements are arranged across the molecular scaffold. This is a chemically meaningful result, because effective CA inhibition likely depends on more than local zinc-binding-group character alone; it also requires a scaffold-level organization that can support catalytic-pocket engagement together with favorable outer-pocket or entrance-region complementarity.

The recurrent appearance of SpMax2_Bh(*), GATS*, MATS*, and VE1* descriptors further indicates that global topology, scaffold size/branching, and distributed physicochemical patterning contribute materially to predictive performance. These descriptor families summarize how mass, volume, electronegativity, polarizability, and graph connectivity are propagated across the molecular structure, thereby capturing global scaffold properties that cannot be reduced to a single fragment or functional group. Their persistence across both training and test subsets suggests that the model is learning a stable integration of local inhibitory motifs with whole-scaffold context, rather than relying on a narrowly defined structural alert.

This concordance between PFI and SHAP, together with the train-test stability of the highest-ranking descriptors, supports the conclusion that the final model is driven by a chemically coherent and transferable descriptor signal within the CAI chemical space investigated. This is an important result for the practical use of the model: it indicates that the predictions are not only quantitatively useful but also interpretable, informing downstream medicinal chemistry reasoning and hit prioritization.

### 2.4. Prospective Virtual Screening and External Experimental Validation Using PubChem CAI K_i_ Records

To extend the learned CAI structure–activity relationship beyond the ChEMBL training distribution while remaining within a chemically relevant neighborhood of potent inhibitors, we performed a PubChem 2D similarity expansion using the most potent ChEMBL CAI compounds $\left(K_{i}\leq 10\;\mathrm{nM};\;pK_{i}\geq 8\right)$ as seed structures. Applying a stringent 2D similarity threshold (Tanimoto ≥ 0.90) retrieved 25,315 unique PubChem analogs, thereby generating a focused near-neighbor chemical-space expansion around high-activity CAI chemotypes. All retrieved compounds were processed through the same AlvaDesc-2D descriptor-generation and leakage-controlled preprocessing pipeline established for the ChEMBL dataset, ensuring that external inference was performed within a strictly consistent descriptor framework. Application of the finalized bagging QSAR model to this expanded set, using a high-potency prioritization threshold of predicted $pK_{i}\geq 8$, yielded 233 high-priority candidates.

To assess predictive transfer beyond the internal ChEMBL train/test split, these 233 QSAR-prioritized PubChem candidates were cross-checked against PubChem bioassay and target annotations for CAI-specific inhibition constant $\left(K_{i}\right)$ measurements mapped to human CAI (UniProt P00915). Under the predefined curation criteria, the set partitioned into 145 compounds with retrievable experimental CAI $K_{i}$ values (Supplementary Table S1) and 88 compounds without retrievable qualifying CAI $K_{i}$ records (Supplementary Table S2). Importantly, the latter group was not treated as inactive compounds; rather, these compounds lacked suitable CAI-specific $K_{i}$ records that met the inclusion criteria used for external validation. For the experimentally annotated subset, $K_{i}$ values reported in µM were converted to an experimental potency scale consistent with the QSAR endpoint using $pK_{i}^{exp}=6-{log}_{10}(K_{i}[\mu M])$. This transformation is numerically equivalent to the nM-based definition used during model development. When multiple qualifying CAI $K_{i}$ records were available for the same PubChem CID, the median value was retained as a robust summary statistic for validation, while the corresponding minimum, maximum, and record-count information were preserved in Supplementary Table S1.

**Figure 5:**
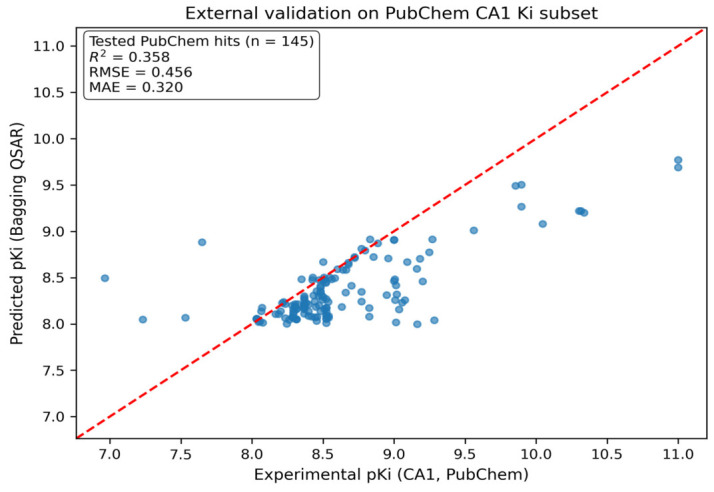
External validation of the bagging QSAR model using PubChem CAI inhibition constants. Scatter plot of experimental $pK_{i}$ values (x-axis; derived from PubChem Ki in µM as $pK_{i}^{exp}=6-{log}_{10}(K_{i}[\mu M])$) versus predicted $pK_{i}^{pred}$ values (y-axis) for the subset of PubChem-screened hits with available CAI *K_i_* measurements (*n* = 145). The red dashed line indicates identity (Predicted = Experimental).

The relationship between predicted and experimental potencies for the 145 externally validated compounds is shown in Figure 5. On this independent PubChem-derived subset, the model achieved $R^{2}=0.358$ with RMSE = 0.456 and MAE = 0.320, demonstrating a meaningful external predictive signal under independent assay provenance. Although this performance is lower than that observed on the internally held-out ChEMBL test split, it is both expected and informative in the present setting: PubChem-derived validation introduces assay heterogeneity, inter-laboratory variability, and reporting noise, and the similarity-expanded compounds—while constrained by high 2D similarity—still represent a modest chemical-space shift relative to the ChEMBL-derived training distribution. Accordingly, this validation should not be framed as unrestricted scaffold extrapolation; rather, it supports near-neighbor generalization in a high-similarity external regime, which is an appropriate and meaningful claim for a model intended for prospective enrichment and prioritization rather than replacement of standardized kinetic characterization. In practical terms, an RMSE of ~0.46 $pK_{i}$ units indicates that predictions typically deviate by <0.5 log units on this external subset, a level of error commonly sufficient for ranking and enrichment in public-database screening workflows.

Figure 5 also illustrates the model’s behavior at the upper potency tail: highly potent compounds remain strongly prioritized even when predicted values show mild regression-to-the-mean, which is typical of continuous QSAR models. For example, PubChem CID 122187164 exhibited an experimental potency of $pK_{i}=11.0$, whereas the model predicted $pK_{i}=9.77$. While this reflects conservative underestimation at the extreme end, the compound remains among the top-ranked candidates—an operationally more important outcome in prospective screening than exact recovery of extreme values.

Finally, the 88 predicted hits without retrievable CAI *K_i_* records constitute the most prospective component of the PubChem screen under current public data coverage. Because these compounds cannot be benchmarked against available CAI experimental measurements, they were advanced to coordination-aware docking and SwissADME profiling as novel CAI inhibitor hypotheses requiring further structural and developability-based triage prior to experimental confirmation.

### 2.5. Coordination-Aware Docking of QSAR-Prioritized Novel Candidates

The 88 QSAR-prioritized hits without retrievable CAI $K_{i}$ records (Supplementary Table S2) represent the genuinely prospective component of the PubChem screening stage. From this set, we selected ten non-duplicate, structurally diverse candidates for structure-based triage to test whether ligand-based prioritization can be supported by catalytically plausible CAI binding geometries (Table 3). The docking panel was deliberately designed to span mechanistically distinct chemotypes rather than to over-sample a single scaffold family; it includes:

(i) Classical sulfonyl Zn-binding candidates (N-alkyl sulfamides and related sulfonyl-rich scaffolds);
(ii) Charged/heteroaromatic sulfonamide–thiadiazole motifs expected to engage the entrance region via electrostatics, and;
(iii) Non-classical O-donor probes—a halogenated carboxylic acid and a tetralin–urea/phenol—to stress-test QSAR extrapolation beyond canonical sulfonamide-centered chemistry.

This diversity-oriented selection is important because it allows docking to function as a mechanistic stress test of whether the QSAR model generalizes across alternative CAI-relevant binding hypotheses while preserving structural novelty.

As summarized in Table 3, the chosen ligands were not simply the “top 10 by score,” but were selected to balance predicted potency, scaffold diversity, and mechanistic breadth. For example, the methoxy-substituted N-alkyl sulfamides (CID 103935964 and 112684680) probe whether subtle electronic/steric tuning (ortho vs. 3,4-dimethoxy) preserves a coordination-consistent Zn-proximal pose, while the tertiary sulfamide (CID 58923319) challenges protomer sensitivity and steric tolerance. The tail-extended sulfamide (CID 72793783) explicitly maps channel/entrance tolerance via a flexible carbamate–benzyl extension, whereas the bulky coumarin–thiadiazole sulfonamide hybrid (CID 101018082) probes whether CAI can accommodate multi-point recognition within the catalytic cone and rim. The inclusion of a permanently charged pyridinium sulfonamide–thiadiazole (CID 118731313) and a highly functionalized thiadiazole–sulfonyl scaffold with a thiocarbamoyl–morpholine tail (CID 74984350) further expands the panel toward high-polarity, multi-contact motifs that may favor electrostatic steering and rim solvation matching. Finally, the halogenated aryl–cyclopropyl carboxylic acid (CID 122367674) serves as a non-sulfonamide control to test whether an O-donor can achieve Zn proximity and whether halogen-driven complementarity stabilizes a plausible binding hypothesis, while the tetralin–urea/phenol (CID 132523446) probes entrance/rim stabilization as a non-classical mode.

Docking outcomes were interpreted using a geometry-first (coordination-aware) framework rather than a score-first approach. Because generic docking scores may under-represent zinc-coordination energetics, retained poses were filtered using an explicit plausibility criterion requiring Zn-N or Zn-O ≤ 2.6 Å to a chemically credible donor atom. In this workflow, the 2.6 Å cutoff was treated as a conservative crystallographically plausible threshold within the CAI active-site context, not as a literal bond-length definition. This represents a key strength of the present approach: docking is used primarily to separate coordination-consistent catalytic-pocket poses from rim-binding or non-canonical alternatives, instead of being interpreted as a direct surrogate for affinity. This framing is especially appropriate for CAI, where direct Zn anchoring remains the canonical inhibition mode, yet non-classical or entrance/rim-biased engagement may still occur depending on scaffold architecture and protonation state.

Within this interpretation scheme, the sulfur-rich ligands in Table 3 function as informative probes of model plausibility: recovery of coordination-consistent poses provides structural support that the QSAR model is capturing chemically meaningful CAI inhibitor features beyond statistical enrichment of sulfur-containing motifs. Conversely, the inclusion of the carboxylate and phenolic candidates provides a useful boundary test: failure to achieve canonical Zn anchoring remains informative because it helps classify these scaffolds as likely entrance/rim binders under the docking model, while still preserving them as experimentally testable hypotheses (potentially with non-classical mechanisms). Overall, this docking stage should be interpreted as mechanistic triage rather than potency proof: it adds a structural plausibility layer to the ligand-based screen, helping prioritize candidates that are both QSAR-supported and structurally credible enough to motivate experimental follow-up.

### 2.6. Structure-Based Triage of Ten Novel QSAR Leads by Coordination-Aware Docking (SwissDock–Vina; CAI PDB: 1AZM)

To provide structure-based support for the ten QSAR-prioritized PubChem leads lacking CAI $K_{i}$ records, each ligand was docked against CAI (PDB: 1AZM) in both its neutral and chemically relevant anionic/protomeric forms (20 dockings total) using a Zn-centered search space. Because empirical Vina scores may be unreliable for zinc metalloenzymes, docking outcomes were interpreted using a geometry-first rule. Poses were classified as coordination-consistent only when they satisfied Zn-N ≤ 2.6 Å for sulfonamide/sulfamide-type N-donor chemotypes or Zn-O ≤ 2.6 Å for O-donor chemotypes. This cutoff was applied as a conservative crystallographically plausible filter in the CAI active-site context, rather than as a literal bond-length definition. When neither criterion was met, the closest-to-Zn pose was still reported for transparency but was interpreted as a rim/entrance-binding or non-canonical Zn-proximal hypothesis rather than as a classical Zn-anchored inhibition mode (Supplementary Table S3).

Overall docking outcome distribution: Across the 20 ligand/protomer docking runs, 12/20 yielded a canonical Zn-anchored pose (10 Zn-N and 2 Zn-O), 1/20 produced a borderline Zn-N interaction $\left(2.6–3.0\;Å\right)$, 3/20 were classified as non-canonical Zn-O-proximal solutions lacking corresponding Zn-N anchoring, and 4/20 remained rim/entrance-biased or off-Zn even after tight-box redocking where indicated. The associated selected docking energies spanned a broad but interpretable range, from approximately −8.32 to −5.08 kcal/mol among the coordination-consistent solutions, underscoring that nominal score alone did not determine mechanistic credibility. Taken together, this outcome distribution shows that the docking stage contributed meaningful mechanistic triage beyond simple score ranking by separating coordination-consistent catalytic-pocket hypotheses from rim-biased and non-canonical alternatives.

High-confidence Zn-anchored binders (canonical coordination recovered): Several chemotypes reproducibly formed Zn-anchored poses with Zn-donor distances in the canonical range. The bulky coumarin-thiadiazole sulfonamide hybrid (CID 101018082) was the most structurally supported case, achieving Mode 1 Zn-N anchoring in both protomers with strong Vina affinities $\left(\Delta G\approx -8.32\;\mathrm{and}\;-8.25\;\mathrm{kcal}/\mathrm{mol};\;\mathrm{Zn}\text{-}N=2.42/2.39\;Å\right)$. The aryl sulfonamide-thiadiazole sulfamide carrying a thiocarbamoyl-morpholine tail (CID 74984350) likewise recovered canonical Zn-N anchoring in both forms $\left(\mathrm{Zn}\text{-}N=2.46/2.40\;Å;\;\mathrm{selected}\;\Delta G\approx -6.81/-7.26\;\mathrm{kcal}/\mathrm{mol}\right)$. In addition, CID 118731313 and CID 136048763 yielded canonical Zn-N-anchored solutions in both protomeric states, with Zn-N distances spanning approximately 2.31–2.59 Å, thereby supporting zinc-site plausibility for these sulfur-rich scaffolds under the current docking model. Collectively, these cases define the most coordination-consistent subset in Table S3 and provide the strongest structure-based support for advancing these chemotypes as classical Zn-anchored CAI inhibitor hypotheses.

Protomer sensitivity and “score–geometry decoupling” (why the geometry filter matters): Several ligands showed a recurring docking behavior in which the best-scoring Vina pose (Mode 1) was not the most chemically credible solution with respect to catalytic zinc coordination. Instead, a lower-ranked pose often fulfilled the predefined Zn-donor geometry filter. This effect was especially clear for CID 118731313, where the selected Zn-N-anchored poses were associated with ΔG≈−6.72/−6.81 kcal/mol, while the corresponding Mode 1 poses had slightly more favorable nominal scores $\left(\approx -7.07/-7.10\;\mathrm{kcal}/\mathrm{mol}\right)$ but remained off-Zn. An analogous pattern was observed for CID 136048763, where the selected Zn-N-anchored poses occurred at ΔG≈−6.58/−6.35 kcal/mol, whereas the top-ranked Mode 1 poses $\left(\approx -7.13/-7.66\;\mathrm{kcal}/\mathrm{mol}\right)$ failed to satisfy the zinc-coordination criterion. These observations justify reporting both the selected (geometry-filtered) and best-score poses and underscore a central principle of the present docking workflow: in a zinc-metalloenzyme setting, pose plausibility must take precedence over nominal docking score when the two are discordant.

Borderline/non-canonical coordination and rim-biased ligands (mechanistic alternatives): Three motifs warrant more cautious interpretation:

- CID 103935964 (o-methoxy aryl-alkyl sulfamide): the neutral form achieved canonical Zn–N anchoring (Zn–N 2.37 Å), whereas the anion returned only borderline Zn–N proximity (Zn–N 2.71 Å), suggesting either protomer sensitivity or an automated-pipeline limitation for this small sulfamide under the selected settings.
- CID 112684680 (3,4-dimethoxy aryl-alkyl sulfamide): the neutral form recovered canonical Zn–N anchoring (Zn–N 2.59 Å), but the anion did not (Zn–N 4.20 Å) and instead showed non-canonical Zn–O proximity (Zn–O 2.29 Å), consistent with a “Zn–O-prox without Zn–N” class that should be treated as a hypothesis rather than a confirmed zinc-binding geometry.
- CID 58923319 (tertiary sulfamide): the anion produced Zn–O-prox only (Zn–O 2.51 Å, Zn–N 3.57 Å) and the neutral remained off-Zn, consistent with the expectation that tertiary substitution/protomer assignment can disrupt classical Zn–N anchoring in automated docking workflows.

Finally, two ligands behaved as rim/entrance-biased under Vina: the tail-extended sulfamide (CID 72793783) remained non-anchored even after tight-box redocking (anion: Zn–O 2.49 Å but Zn–N 4.52 Å; neutral: Zn–O 2.75 Å, Zn–N 8.00 Å), and the tetralin–urea/phenol (CID 132523446) failed to reach Zn–O ≤ 2.6 Å in both neutral and phenolate forms (Zn–O 3.43/3.21 Å), supporting an entrance/rim binding hypothesis rather than direct Zn coordination.

O-donor control (carboxylic acid) behaves as expected geometrically: The dibrominated aryl–cyclopropyl carboxylic acid (CID 122367674) achieved canonical Zn–O anchoring in both forms (Zn–O 2.31/2.40 Å), but its best-score Mode 1 remained off-Zn, again illustrating score–geometry decoupling and supporting the choice to treat docking primarily as a coordination plausibility filter rather than an affinity estimator.

Taken together, Table S3 supports prioritizing ligands that (i) reproducibly recover canonical Zn anchoring across protomers and (ii) do so without requiring “rescue” by extreme box constraints—most notably 101018082, 74984350, and the consistently Zn-anchored sulfonyl-rich scaffolds (118731313, 136048763), alongside the O-donor control 122367674 for mechanistic breadth. Conversely, 72793783 and 132523446 should be carried forward, if at all, explicitly as rim/entrance binding hypotheses, while 58923319/112684680 (anion) are best reported as non-canonical Zn-prox cases that document known limitations of automated Vina pipelines for zinc coordination.

### 2.7. Structure–Activity Relationships of QSAR-Prioritized CAI Leads

To rationalize why certain QSAR-prioritized candidates constitute more credible CAI inhibitor hypotheses than others, we integrated three orthogonal evidence layers: predicted potency ($pK_{i}^{pred}$), chemotype/ZBG class (Table 3), and coordination-aware docking outcomes (Supplementary Table S3). This SAR analysis is deliberately conservative and reviewer-robust: docking is treated strictly as a pose-plausibility filter under an explicit zinc-geometry rule (Zn–N ≤ 2.6 Å for N-donor chemotypes; Zn–O ≤ 2.6 Å for O-donor chemotypes), rather than as a stand-alone affinity estimator. Accordingly, the key SAR question is not “which ligand scores best,” but which ligand can realize a chemically credible CAI binding hypothesis consistent with its ZBG identity and microstate.

- ZBG identity and accessibility define the primary SAR axis: canonical Zn anchoring vs. alternative modes: Across the docked set, the strongest structural support was observed for chemotypes capable of presenting a binding-competent N-donor ZBG toward the catalytic Zn^2+^ center, consistent with classical CA inhibition. Several sulfur-rich scaffolds—most notably CIDs 101018082, 118731313, 74984350, and 136048763—recovered canonical Zn–N anchoring under the geometry filter, supporting their interpretation as high-confidence Zn-anchored CAI inhibitor hypotheses. In contrast, candidates that failed to satisfy the Zn–donor threshold even after tight-box resampling were most plausibly interpreted as rim/entrance-biased binders or non-canonical Zn-proximal solutions. These outcomes remain mechanistically informative (and valuable for hypothesis testing), but they should be framed explicitly as non-canonical until biochemical data confirms an inhibition mechanism.
- Protomer sensitivity emerges as a secondary SAR layer within “favorable” ZBG classes: Even among sulfamide/sulfonamide-like chemotypes, canonical Zn anchoring proved microstate-dependent, which is an important practical SAR nuance for CAI. Local substitution patterns and scaffold microenvironment can modulate whether the ZBG is presented in a docking-competent orientation. Notably, the compact N-alkyl sulfamide-like candidates CID 103935964 and CID 112684680 achieved Zn–N anchoring predominantly in their neutral forms, whereas the corresponding anionic protomers shifted toward borderline or non-canonical Zn proximity. This pattern emphasizes a critical design lesson: ZBG presence is necessary but not sufficient—ZBG accessibility and protomer correctness are decisive for realizing a canonical Zn-anchored hypothesis and for determining whether a compound should be progressed as a classical Zn binder or treated as protomer-sensitive.
- N-alkylation at the sulfamide nitrogen marks a practical SAR boundary: The tertiary sulfamide CID 58923319 represents a boundary case. It failed to recover a canonical Zn–N pose in either protomer (Zn–N > 3.5 Å), yielding only Zn–O-proximal or off-Zn solutions despite a high $pK_{i}^{pred}$. This is SAR-informative rather than anomalous: N-alkylation can impede productive Zn^2+^ coordination and can increase sensitivity to atom typing and charge assignment in automated docking pipelines. Consequently, tertiary-sulfamide scaffolds are best treated as non-canonical binding hypotheses until biochemical data clarify their inhibition mechanism and ionization dependence.
- O-donor chemotypes act as mechanistic probes and QSAR extrapolation controls: The non-sulfonamide dibrominated aryl–cyclopropyl carboxylic acid CID 122367674 reproducibly achieved canonical Zn–O proximity (≈2.3–2.4 Å) in both acid and carboxylate forms, supporting the plausibility of an O-donor anchoring mode in CAI and serving as a useful QSAR extrapolation control beyond classical sulfonamide space. By contrast, the tetralin–urea/phenol CID 132523446 and the tail-extended sulfamide CID 72793783 did not meet Zn–donor ≤ 2.6 Å criteria even after focused resampling, favoring rim/entrance-biased solutions under the current docking model. These compounds therefore remain valuable as alternative-mechanism probes (e.g., entrance/cap stabilization) rather than as canonical Zn-anchored inhibitors.

Importantly, these SAR patterns are concordant with the QSAR explainability profile. Descriptor families associated with sulfur/heteroatom connectivity and polar–lipophilic organization dominate attribution, implying that the model responds not only to a ZBG-like signature but also to how that functionality is embedded and organized along the scaffold—a descriptor-level proxy for zinc accessibility coupled with entrance/cone complementarity. This agreement between descriptor-level explanation and coordination-aware docking strengthens confidence that prioritization is driven by reproducible, chemically plausible structure–activity signal rather than by model artifacts. Table 4 summarizes the integrated SAR interpretation linking $pK_{i}^{pred}$, ZBG class, docking-supported binding mode, protomer sensitivity, and the resulting triage tier.

### 2.8. Pharmacokinetic Profiling and ADME Prediction Using SwissADME

While high predicted inhibitory potency ($pK_{i}^{pred}$) and coordination-consistent docking poses support prospective CAI engagement, progression toward experimentally testable leads requires a favorable balance between potency, mechanistic plausibility, and developability. Accordingly, the ten QSAR-prioritized and docked candidates were profiled using SwissADME to contextualize their physicochemical space, oral drug-likeness, and key ADME-related liabilities (Supplementary Table S4).

- Physicochemical space and drug-likeness: Overall, the set occupies a drug-like yet intentionally diverse property space, spanning MW 230–542 Da, TPSA 52.6–251.4 Å^2^, and consensus logP −0.65 to 3.21. This breadth reflects deliberate inclusion of both compact sulfamide/sulfonamide chemotypes, and larger sulfur-rich scaffolds designed to probe the catalytic cone and entrance/rim complementarity observed in the docking/SAR analyses. Drug-likeness compliance was generally favorable: 8/10 compounds showed zero Lipinski violations, whereas 2/10 displayed two violations each, primarily driven by elevated polarity and heteroatom density. Importantly, these higher-complexity outliers also exhibited the least favorable absorption proxies (lower predicted GI absorption and reduced SwissADME bioavailability scores), supporting internal consistency across the developability readouts.
- Solubility and early developability: Solubility predictions from multiple SwissADME estimators (ESOL, Ali, and Silicos-IT) indicate broadly tractable aqueous behavior at the screening stage. Most compounds fell into soluble to moderately soluble classes, with a subset predicted to be very soluble, and only a minority trending toward poor solubility depending on the estimator. This is practically relevant for early enzymatic testing: maintaining reasonable solubility reduces formulation confounding and supports interpretable concentration–response characterization. Where solubility is predicted to be borderline (typically in higher-logP or larger scaffolds), prioritization should favor early confirmation using experimental solubility/permeability triage rather than late-stage attrition.
- Liability screening: PAINS/Brenk, absorption-distribution, and transport: From an assay-liability perspective, no PAINS alerts were detected (0/10), reducing concern that the predicted activities are driven by classical interference motifs. Brenk alerts were observed for a minority of compounds (4/10) and appeared to be substructure-specific rather than pervasive, supporting a compound-by-compound assessment rather than blanket exclusion. These filters were therefore used as early liability indicators within the triage framework, rather than as automatic exclusion criteria.
- The BOILED-Egg model further refined translational expectations: Overall, 6/10 compounds were predicted to have high GI absorption, supporting oral exposure potential for a majority of the set. In parallel, 7/10 were predicted to be non-BBB permeant, a potentially advantageous profile for systemic CA modulation when minimizing unintended CNS exposure is preferred. Transporter-related risk was low overall, with 9/10 predicted not to be P-glycoprotein substrates, suggesting limited efflux-driven bioavailability concerns for most candidates.
- Metabolic interaction flags and practical next steps: Predicted metabolic interaction risk was moderate and scaffold dependent. CYP inhibition alerts were most frequent for CYP2C19 (4/10), followed by CYP2C9 (2/10) and CYP3A4 (2/10); CYP1A2 inhibition was rare (1/10) and CYP2D6 inhibition was not predicted (0/10). These patterns suggest that most candidates fall within an acceptable early liability window, but a subset—particularly higher-TPSA, multi-sulfonyl or more complex scaffolds with multiple CYP flags—should be advanced cautiously. For those candidates, early follow-up should prioritize permeability assessment, metabolic stability, and DDI-risk screening alongside CA isoform profiling to ensure that improved potency does not translate into avoidable developability penalties.

Collectively, SwissADME profiling indicates that most QSAR-prioritized and docking-supported CAI leads occupy a practically developable region of chemical space suitable for progression from virtual prioritization to biochemical testing. At the same time, the results delineate clear, structure-dependent ADME trade-offs—especially polarity-driven absorption limits and CYP-flag clustering—enabling rational triage rather than post hoc attrition. This supports the value of embedding ADME assessment as a downstream—but decisive—filter in the integrated CAI discovery workflow.

## 3. Discussion

### 3.1. Predictive Performance and Model Selection for Deployable Screening

This study was designed to deliver a deployable and interpretable QSAR pipeline for CAI potency prediction rather than a model optimized solely for internal fit. Across five regressors trained under identical, leakage-controlled preprocessing and feature selection, the key discriminator was held-out test generalization. The bagging ensemble provided the best out-of-sample performance (test $R^{2}=0.646$; RMSE ≈ 0.61; MAE ≈ 0.45), marginally outperforming GB and XGB on test $R^{2}$ despite their stronger training fits. This ranking is consistent with bagging’s bias–variance behavior in noisy, high-dimensional descriptor spaces: it tends to be robust when the intended use-case is prospective prioritization under chemical-space shift, where avoiding brittle overfitting is more important than minimizing training error.

### 3.2. What the QSAR Learned: Zinc-Binding Competence Plus Scaffold-Level Context

The descriptor-reduction and interpretation results support a chemically coherent message: CAI potency is not explained by a single functional-group “trigger,” but by the combination of (i) sulfur/heteroatom connectivity patterns that often accompany CA inhibitor chemotypes, and (ii) scaffold-level context that governs orientation, pocket complementarity, and entrance/rim interactions. The dominant descriptor families captured (a) sulfonyl-adjacent environments, (b) polar–lipophilic organization (pharmacophore-like 2D patterning), and (c) global topology/shape and distributed-property autocorrelations that proxy size/branching and electronic/steric propagation through the molecular graph. Mechanistically, this is an expected signature for CA inhibition: Zn-site engagement is often necessary, but potency depends on how the scaffold packs into the CAI cone and stabilizes secondary contacts.

### 3.3. XAI Concordance: Stability of Feature Signal Across Training and Test

PFI and SHAP were used as complementary explainability lenses to test whether the learned signal is stable rather than split-specific. Both were reported as absolute mean magnitudes, quantifying contribution strength rather than direction. The key result was concordance: the same descriptor families remained prominent across training and test, with the expected attenuation of magnitudes on test. This agreement supports the conclusion that the bagging model relies on reproducible structure–activity signal rather than idiosyncratic correlations, while motivating interpretation at the descriptor-family level because partial collinearity can persist even after correlation pruning.

### 3.4. Prospective PubChem Screening and External Experimental Validation

A major strength of the work is evaluation beyond the ChEMBL split via a similarity-guided expansion into PubChem. Starting from potent ChEMBL inhibitors (*K_i_* ≤ 10 nM; *pK_i_* ≥ 8), a stringent 90% 2D similarity search retrieved 25,315 PubChem analogs. Applying the finalized bagging QSAR and using a high-potency cutoff ($pK_{i}^{pred}$ ≥ 8.0) yielded 233 prioritized candidates. PubChem annotations enabled retrospective mapping of experimental CAI values for 145 of these hits (median used when multiple records existed), providing an external validation set. On this independent set, the model achieved R^2^ = 0.358, RMSE = 0.456, and MAE = 0.320—an error profile that is decision-useful for ranking/enrichment in screening, especially given heterogeneous assay provenance and residual distribution shift even under high similarity constraints. Importantly, because this validation is a near-neighbor expansion, it supports transferability within adjacent chemical space rather than unconstrained scaffold hopping.

### 3.5. Docking as Mechanistic Triage, Strengthened by SAR Integration

For zinc metalloenzymes, docking must be interpreted cautiously because generic scoring functions can under-represent coordination energetics and can rank off-metal poses too favorably. Accordingly, docking here was used as a mechanistic plausibility filter: each of the ten QSAR-prioritized ligands was docked in neutral and relevant anionic/protomer states (20 runs total), and pose selection was enforced by zinc geometry (Zn–N ≤ 2.6 Å for N-donor chemotypes; Zn–O ≤ 2.6 Å for O-donor chemotypes). When canonical anchoring was not achieved, the closest-to-Zn pose was reported and explicitly flagged.

The integrated SAR analysis (Table 4) clarifies why docking outcomes diverged among high-$pK_{i}^{pred}$ candidates:

- ZBG identity and accessibility dominate: sulfur-rich N-donor chemotypes most consistently recovered canonical Zn–N anchoring (e.g., CIDs 101018082, 118731313, 74984350, 136048763), supporting classical CA-binding plausibility.
- Protomer sensitivity is a second SAR layer: compact N-alkyl sulfamide-like leads (CIDs 103935964 and 112684680) achieved Zn–N anchoring primarily in the neutral form, whereas anionic protomers shifted toward borderline or non-canonical proximity—highlighting that “ZBG present” is insufficient unless the microstate and presentation are compatible.
- A practical boundary case: the tertiary sulfamide CID 58923319 did not recover canonical Zn–N anchoring (Zn–N > 3.5 Å), motivating its treatment as a non-canonical/off-Zn hypothesis pending biochemical confirmation.
- O-donor controls matter: CID 122367674 reproducibly achieved Zn–O anchoring and therefore serves as a high-value mechanistic probe and QSAR extrapolation test beyond sulfonamide-like space.

These SAR-linked interpretations make the docking layer reviewer-proof: docking is not used to “prove” affinity, but to discriminate coordination-credible versus rim/entrance-biased hypotheses within QSAR-selected candidates.

### 3.6. Developability and ADME Implications from SwissADME

SwissADME profiling adds a necessary developability lens to the QSAR + docking shortlist. The ten ligands span a practical property range (MW 230–542 Da; TPSA 52.6–251.4 Å^2^; consensus logP −0.65 to 3.21) and are not dominated by obvious assay-interference liabilities (PAINS = 0/10). Lipinski compliance was generally favorable (8/10 with zero violations), while 2/10 showed two violations consistent with higher polarity and heteroatom-rich scaffolds. Absorption/distribution predictions stratified the set: 6/10 were predicted to have high GI absorption, and 7/10 were predicted to be non-BBB permeant (often favorable when CNS exposure is not desired). P-gp substrate risk was low overall (9/10 predicted non-substrates). CYP inhibition flags were present but concentrated (most frequently CYP2C19), supporting compound-specific caution and early DDI-risk screening for flagged scaffolds. A practical trade-off emerges: some of the most coordination-credible, sulfonyl-rich scaffolds are also among the most polar and thus show lower predicted oral absorption—positioning them as strong biochemical tools or optimization starting points rather than immediate oral leads.

### 3.7. Integrated Prioritization: Tiered Progression of the Ten Prospective CAI Leads

The central outcome of this study is a decision-ready, tiered prioritization of ten prospective CAI inhibitor hypotheses (Table 4), derived from the convergence of three orthogonal evidence layers: (i) QSAR-predicted potency, (ii) coordination-aware docking plausibility under an explicit Zn–donor geometry rule (or an explicitly stated alternative binding hypothesis when canonical anchoring is not achieved), and (iii) early developability indicators from SwissADME. This framework is intentionally designed to be transparent, mechanistically defensible, and experimentally testable: it avoids over-interpretation of docking scores in a zinc metalloenzyme context and prevents potency-only ranking from advancing chemotypes with poor structural plausibility or impractical developability. Rather than producing a single “top hit,” the workflow provides an actionable progression logic that connects model predictions to concrete next experiments and go/no-go criteria.

Tier 1—prioritize first for biochemical testing (highest-priority computational hypotheses): CIDs 103935964, 112684680, 122367674. Tier 1 contains candidates that combine high predicted potency with canonical (or near-canonical) Zn-site plausibility and manageable ADME flags, making them the most suitable for immediate CAI biochemical testing.

- CID 103935964 (N-alkyl sulfamide; $pK_{i}^{pred}=9.01$) achieved Zn–N anchoring in the neutral protomer (with borderline anchoring in the anion), consistent with a classical Zn-binding hypothesis and highlighting protomer sensitivity as a practical experimental consideration. Its SwissADME profile is favorable (no PAINS; generally drug-like), supporting rapid progression.
- CID 112684680 (dimethoxy N-alkyl sulfamide; $pK_{i}^{pred}=8.55$) recovered canonical Zn–N anchoring in the updated neutral docking run and maintains a developability profile compatible with near-term enzymatic validation.
- CID 122367674 (dibrominated aryl–cyclopropyl carboxylic acid; $pK_{i}^{pred}=8.91$) achieved canonical Zn–O anchoring in both acid/carboxylate representations. Importantly, it also functions as a high-value mechanistic probe and QSAR extrapolation control beyond sulfonamide/sulfamide space; its inclusion strengthens the manuscript’s credibility by explicitly testing whether the workflow can prioritize a plausible O-donor chemotype.

Tier experimental implication: Tier 1 should be prioritized for immediate CAI inhibition assays (e.g., stopped-flow CO_2_ hydration or an equivalent kinetic readout), with explicit tracking of pH and ionic strength and, where feasible, testing of relevant microstates (neutral vs. deprotonated) for protomer-sensitive ligands.

Tier 2—test in parallel (coordination-credible but with developability liabilities or mechanistic sensitivity): CIDs 101018082, 118731313, 74984350, 136048763. Tier 2 includes candidates that satisfy coordination-credible Zn anchoring but display higher polarity/complexity or absorption/distribution flags, positioning them as strong biochemical leads and optimization starting points rather than immediate oral lead prototypes.

- CID 101018082 (bulky coumarin–thiadiazole sulfonamide hybrid; $pK_{i}^{pred}=8.46$) showed strong Zn–N anchoring and represents a multi-point recognition scaffold that may engage both the catalytic cone and rim; its size/polarity warrants developability monitoring.
- CID 74984350 (sulfonyl/thiadiazole-rich scaffold with thiocarbamoyl–morpholine tail; $pK_{i}^{pred}=8.41$) recovered canonical Zn–N anchoring and is mechanistically informative for mapping rim solvation and H-bonding capacity, but its higher polarity/complexity suggests it is best advanced as an optimization scaffold.
- CID 118731313 (cationic pyridinium sulfonamide–thiadiazole; $pK_{i}^{pred}=8.30$) achieved Zn–N anchoring but may be influenced by entrance electrostatics; it should be evaluated with care because score–geometry decoupling is common for charged ligands in generic docking.
- CID 136048763 (highly polar dual-sulfonyl thioheterocycle; $pK_{i}^{pred}=8.14$) recovered Zn–N anchoring yet occupies a high-polarity regime; it functions as a stringent stress-test for CAI binding and a useful baseline for property-driven analog design.

Experimental implication: Tier 2 should be tested alongside Tier 1, but with an explicit “property-aware” plan: early solubility/permeability triage and CA isoform counterscreens (at minimum CA II) to determine whether these scaffolds are best pursued as selective leads or as mechanistic tools.

Tier 3—alternative binding hypotheses (exploratory mechanistic probes): CIDs 72793783, 132523446, 58923319. Tier 3 contains candidates for which docking does not support canonical Zn anchoring in either protomer (or indicates non-canonical Zn proximity). These compounds remain valuable—particularly for exploring rim/entrance binding and non-classical inhibition—but should not be presented as classical Zn-anchored inhibitors without biochemical confirmation.

- CID 72793783 (tail-extended sulfamide; $pK_{i}^{pred}=8.30$) repeatedly preferred rim/entrance poses and failed to achieve canonical Zn anchoring even after tight-box redocking, supporting an entrance-pocket hypothesis.
- CID 132523446 (tetralin–urea/phenol; $pK_{i}^{pred}=8.24$) did not achieve Zn–O anchoring even as a phenolate, indicating a predominantly non-classical or rim-biased interaction under the current docking model.
- CID 58923319 (tertiary sulfamide; $pK_{i}^{pred}=8.19$) showed non-canonical zinc proximity (Zn–O without Zn–N anchoring) and strong protomer sensitivity; it is best framed as a mechanistic probe or protomer/typing-sensitive case.

Experimental implication: Tier 3 should be tested with mechanism-discriminating assays (e.g., zinc dependence, pH dependence, competition with classical sulfonamide inhibitors) to determine whether activity arises from rim binding, weak zinc proximity, or assay-context effects.

These findings demonstrate that integrating QSAR-based potency prediction, coordination-aware docking, and early developability profiling provides a practical and decision-oriented framework for CAI hit triage. Importantly, the tiered prioritization scheme makes clear that predicted potency alone is insufficient for advancement: compounds must also satisfy criteria related to zinc-site binding plausibility and property space compatible with experimental tractability. By distinguishing high-confidence Zn-anchored candidates, mechanistically credible but property-limited leads, and alternative binding hypotheses, the workflow transforms computational outputs into a structured and testable experimental roadmap, thereby supporting the rational staging of compound synthesis or procurement and subsequent kinetic evaluation. Nevertheless, the shortlisted compounds should be interpreted as computationally prioritized hypotheses generated by a tiered in silico triage framework, rather than as experimentally confirmed CAI inhibitors.

## 4. Materials and Methods

This section describes the end-to-end computational framework used to generate robust, reproducible, and interpretable predictions of human CAI inhibitory potency. All data acquisition, preprocessing, modeling, and post-model analyses were implemented as traceable, node-based workflows in KNIME Analytics Platform ((version 5.4, KNIME AG, Zurich, Switzerland available at: https://www.knime.com (accessed on 9 May 2026)), enabling full pipeline transparency and consistent application of transforms across datasets. Structural standardization and cheminformatics operations were performed with RDKit (version 5.2.0, RDKit KNIME extension, available at: https://github.com/rdkit/rdkit/releases (accessed on 9 May 2026)), while chemically interpretable 2D molecular descriptors were computed using alvaDesc (version 3.0.8; Alvascience, Milan, Italy; available at: https://www.alvascience.com/alvadesc/ (accessed on 9 May 2026)). To prevent information leakage and to approximate real deployment behavior, all operators requiring parameter estimation (e.g., imputation statistics, variance/correlation filters, normalization parameters, and feature-ranking/selection) were fit exclusively on the training split and then applied unchanged to the held-out test set and external PubChem validation subsets. Bioactivity records were sourced from ChEMBL database ((release 33; available at: https://www.ebi.ac.uk/chembl/ (accessed on 9 May 2026)) using Target ID: CHEMBL261 because it provides standardized target mapping, curated assay metadata, and consistent bioactivity annotations, allowing reproducible extraction and harmonization of inhibition constants ($K_{i}$) across a broad chemical space—an essential prerequisite for rigorous deduplication, unit standardization, and reliable QSAR regression.

### 4.1. Data Acquisition and Curation

Bioactivity records for human carbonic anhydrase I (CAI) were retrieved from ChEMBL using the target identifier CHEMBL261. Only entries reporting an experimentally determined inhibition constant ($K_{i}$) were retained to ensure a consistent kinetic endpoint for regression modeling. All values were harmonized to nanomolar (nM) units, and potency was expressed on a logarithmic scale to stabilize variance and align with standard QSAR practice: ${pK}_{i}$ = 9 − log10($K_{i}$ [nM]). Chemical structures were standardized at the SMILES level and deduplicated to enforce a one-compound/one-response mapping. When multiple activity measurements were associated with the same standardized SMILES, the median value was retained as the representative response for that structure, thereby reducing sensitivity to outliers and partially buffering assay-to-assay variability.

**Figure 6:**
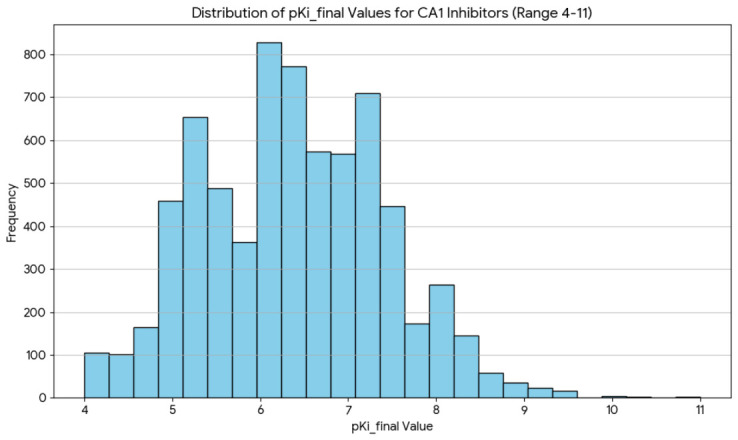
Global distribution of $pK_{i}$ for CAI inhibitors (*n* = 6958).

Records outside the defined modeling domain (${pK}_{i}$ ≥ 4) were excluded to restrict training to an applicability region supported by sufficient signal and to limit extrapolation to weak/uncertain binders. After these curation steps, the dataset comprised 6958 unique CAI inhibitors, reduced from 10,265 $K_{i}$-annotated ChEMBL records. The final curated potency distribution spanned ${pK}_{i}$ = 4–11 (mean ≈ 6.38; median ≈ 6.35; Figure 6). These curated endpoint values, used consistently in all downstream preprocessing, feature selection, model training, and external screening steps, are denoted ${pK}_{i}$_final.

### 4.2. External Chemical-Space Expansion via PubChem Similarity Search

To prospectively explore near-neighbor chemical space around highly potent CAI inhibitors while remaining within a realistic applicability domain of the QSAR, all curated ChEMBL compounds with $K_{i}$ ≤ 10 nM (i.e., ${pK}_{i}$ ≥ 8) were used as seed structures for external expansion. A 2D similarity search was performed against the PubChem Compound repository using a Tanimoto threshold of 0.90, yielding 25,315 unique PubChem CIDs.

Canonical SMILES for the retrieved PubChem compounds were collected and subjected to the same descriptor generation and leakage-controlled preprocessing pipeline used for the ChEMBL dataset (AlvaDesc-2D calculation followed by train-only fitted imputation/filters/normalization, applied unchanged to the external set). This ensured that prospective predictions were produced under the identical feature space and transformation parameters learned from the training subset, enabling direct and unbiased deployment of the trained QSAR model to the PubChem expansion set.

### 4.3. Descriptor Generation

Molecular features were computed using alvaDesc software, restricting descriptor calculation to 2D descriptor blocks to maximize interpretability, computational efficiency, and cross-platform reproducibility [6]. For each molecule, 4224 2D descriptors were generated for both (i) the curated ChEMBL CAI dataset, and (ii) the PubChem similarity expansion set, ensuring that prospective screening was conducted in the same descriptor space used for model development.

2D descriptors were selected because they provide chemically meaningful representations of molecular size, topology, fragment composition, functional-group environments, and atom-centered connectivity patterns, while remaining independent of conformer generation and geometry optimization [27]. This choice reduces methodological variability associated with 3D structure preparation (protonation, conformer ensembles, force fields) and supports rigorous, leakage-controlled preprocessing and feature selection at scale. Moreover, 2D descriptors are well suited to interpretable QSAR, because high-importance variables can be mapped back to recognizable medicinal chemistry motifs (e.g., sulfonyl connectivity, polar–lipophilic organization, and scaffold topology) rather than to opaque latent representations [28].

### 4.4. Leakage-Controlled Preprocessing and Feature Filtering

All preprocessing operations that estimate parameters from data were fit exclusively on the training subset and then applied unchanged to the held-out test subset and the PubChem expansion set. This leakage-controlled design preserves unbiased performance estimation and mirrors the intended deployment scenario in which a model trained once is applied to unseen compounds.

Train/test split: The curated ChEMBL dataset (*n* = 6958) was partitioned into an 80/20 split (training: 80%, test: 20%) using a fixed random seed (reported in the KNIME workflow) to ensure reproducibility.

Missing-value handling and imputation: AlvaDesc encodes non-computable descriptor values using the sentinel −999. These entries were first converted to missing values and then imputed by mean imputation, where descriptor-wise means were computed only on the training set. The learned imputation parameters were subsequently applied unchanged to the test and PubChem sets.

Low-variance filtering: To remove near-constant descriptors with minimal information content, a variance filter was fit on the training set only, retaining descriptors with variance > 0.01. This step reduced the descriptor panel from 4224 to 2548 descriptors. The retained descriptor list was then propagated unchanged to the test and PubChem sets.

Correlation pruning: To reduce redundancy and multicollinearity, an absolute pairwise correlation filter was fit on the training set only using |r| ≥ 0.90 as the exclusion threshold. Redundant descriptors within highly correlated blocks were removed according to the KNIME Cross Correlation Filter node logic. This reduced the descriptor space from 2548 to 1373 descriptors, and the resulting feature set was applied identically to the test and PubChem sets.

Normalization: Descriptor scaling was performed using Z-score normalization (mean-centering and variance scaling). Descriptor-wise and were estimated on the training set only, and the fitted normalization parameters were then applied unchanged to the test and PubChem sets.

### 4.5. Feature Selection by Bagged-Tree Ranking

To obtain a compact, interpretable descriptor panel suitable for both model development and prospective screening, feature ranking was performed using a bagging ensemble of decision trees trained exclusively on the training subset after leakage-controlled preprocessing and filtering (1373 descriptors). Bagging was selected because bootstrap aggregation tends to yield more stable importance estimates in high-dimensional QSAR settings, particularly when descriptors are partially correlated and bioactivity measurements include inter-assay variability. By averaging variable-importance signals across many resampled training subsets, bagging reduces the likelihood that descriptor selection is driven by idiosyncrasies of a single fit [29].

The bagging model was configured with 500 trees, a maximum tree depth of 50, and a row sampling rate of 0.632 (bootstrap-style sampling). Descriptors were ranked according to the bagging-derived importance metric, and the top 100 descriptors were retained as the final feature panel. This fixed top-100 descriptor set was then applied unchanged to the held-out test subset and to the PubChem expansion set for all subsequent model training, test evaluation, and prospective prediction, ensuring that downstream comparisons reflect differences in learning algorithms rather than differences in feature-space definition.

### 4.6. QSAR Model Development and Test-Set Evaluation

Five supervised regression models were implemented in KNIME on the optimized AlvaDesc-2D feature space: Elastic Net (EN) [30], classification and regression tree (CART) [31], bagging ensemble (Bagging) [32], gradient boosting (GB) [33], and eXtreme Gradient Boosting (XGBoost) [34]. All models were trained using an identical, leakage-controlled data pipeline: preprocessing operators (missing-value handling, filtering, and scaling) were fitted on the training subset only and applied unchanged to the held-out test subset and all external sets; model fitting used the fixed top-100 descriptor panel selected from the training subset (Section 3.5). Model performance was evaluated on the held-out test subset using $R^{2}$ and complementary error metrics (RMSE and MAE).

Across algorithms, boosting methods achieved the lowest training error, consistent with their higher model capacity; however, this advantage did not translate into superior out-of-sample performance. The bagging ensemble achieved the strongest test-set generalization and the most stable error profile on unseen data. Given the intended use-case—prospective prioritization under chemical-space variability—model selection emphasized test-set robustness rather than training fit; thus, the bagging model was selected as the primary predictive engine for downstream screening and triage.

### 4.7. Model Interpretability and Explainable AI (PFI and SHAP)

To ensure that predictive accuracy was accompanied by chemically coherent and split-stable signal, we applied two complementary explainability analyses to the selected bagging QSAR model: permutation feature importance (PFI) and SHAP (SHapley Additive exPlanations). PFI provides a model-agnostic, performance-based measure of descriptor relevance by quantifying the drop in predictive performance when a descriptor is randomly permuted while all other variables are held fixed [35,36]. This approach is particularly appropriate for nonlinear ensemble QSAR models and is less susceptible than impurity-based tree importances to biases introduced by split frequency and correlated predictors [37]. In this study, PFI was computed on held-out data to support robustness-focused interpretation and to highlight descriptor families that materially influence predicted CAI potency.

SHAP complements PFI by providing local-to-global interpretability. SHAP values decompose each individual prediction into signed, additive contributions of descriptors relative to a baseline expectation, enabling high-potency predictions to be rationalized in terms of interpretable chemical features rather than treated as opaque outputs [38,39]. Aggregating SHAP values across compounds yields population-level summaries that can be compared directly with PFI rankings, allowing cross-validation of the learned structure–activity signal [37]. Together, PFI (global dependence) and SHAP (directional local explanations) were used to (i) verify that the model relies on mechanistically plausible descriptor families for CA inhibition—most prominently sulfur/heteroatom connectivity and polar–lipophilic organization—, and (ii) assess whether attribution patterns remain stable from training to test partitions. Because descriptor blocks can remain partially correlated even after correlation pruning, importance is interpreted primarily at the descriptor-family level rather than as a unique causal role for any single variable.

### 4.8. Structure-Based Triage by Docking on the SwissDock Platform

To provide structure-based support for QSAR-prioritized candidates and to explicitly assess zinc-site coordination plausibility, the ten structurally diverse leads were evaluated by molecular docking against human CAI using the SwissDock AutoDock Vina service [40]. AutoDock Vina 1.2.x served as the underlying docking engine within this platform. However, in the present workflow, Vina ΔG values were interpreted only in conjunction with the predefined Zn-geometry filter, rather than as standalone evidence of binding plausibility. Accordingly, docking outputs were treated as pose hypotheses rather than definitive affinity estimates, and pose selection was governed by an explicit Zn-donor geometry plausibility rule rather than by Vina score alone.

- Receptor preparation: The crystal structure of human carbonic anhydrase I was retrieved from the Protein Data Bank (PDB: 1AZM) [23]. The co-crystallized ligand and crystallographic waters were removed, while the catalytic Zn^2+^ ion and its coordinating residues (His94, His96, His119) were retained to preserve the native metal-binding environment. Polar hydrogens were added and Gasteiger charges assigned using AutoDockTools (MGLTools), and the receptor was exported in PDBQT format [41]. PDB: 1AZM contains the classical sulfonamide inhibitor acetazolamide bound directly to Zn^2+^ and therefore provides an experimentally resolved reference for the canonical CAI active-site topology: a deep conical catalytic pocket terminating at the zinc center with a solvent-exposed entrance/rim region capable of accommodating substituent extensions.
- Ligand preparation and protomer enumeration: Because carbonic anhydrase inhibition is highly protonation-state dependent, and successful zinc anchoring may require a specific binding-competent microstate, each ligand was prepared in both its neutral and, where appropriate, deprotonated (anionic) forms at approximately physiological pH (∼7.4). This strategy was applied in particular to sulfonamides/sulfamides, carboxylic acids, and phenols, which were represented in both protonation states to minimize false-negative docking interpretations caused by incorrect ionization assumptions and to better capture plausible zinc-binding microstates in the CAI active site [11]. Ligands were subsequently converted into docking-ready formats with standardized hydrogen treatment and charge assignment to support consistent comparison across chemotypes and protomeric forms.
- AutoDock Vina protocol (SwissDck Vina service) and geometry-filtered pose selection: Docking was performed using a Zn-centered search space designed to sample both (i) deep catalytic-pocket poses compatible with zinc anchoring, and (ii) entrance/rim-oriented poses relevant to larger or tail-extended scaffolds. The primary grid box was centered on the catalytic Zn^2+^ ion at $\left(X,Y,Z)=(37.595,16.545,-14.676\right)$ with dimensions of 30 × 30 × 30 Å. For extended ligands that repeatedly localized to peripheral regions, targeted re-docking was additionally carried out using a tighter 20 × 20 × 20 Å box to enforce re-sampling of the catalytic pocket. Docking exhaustiveness was scaled from 20 to 64 according to ligand flexibility, approximated by rotatable-bond burden, to maintain adequate conformational exploration while preserving computational tractability. Because generic docking scores may under-represent transition-metal coordination energetics, poses were not selected on the basis of Vina affinity $\left(\Delta G\right)$ alone. Instead, a coordination-aware zinc-geometry filter was applied as the primary pose-selection rule. In the present workflow, the Zn-donor cutoff of 2.6 Å was used as a conservative crystallographically plausible threshold within the CAI active-site context, rather than as a claim of exact metal-ligand bond geometry. This criterion was intended to distinguish coordination-consistent poses from non-canonical or rim-biased solutions in a zinc metalloenzyme setting, where score alone may favor sterically acceptable but chemically implausible poses. Accordingly, a pose was considered coordination-credible only if the minimum heavy-atom distance satisfied:
  (a) Zn-N ≤ 2.6 Å for N-donor chemotypes (e.g., sulfonamide- or sulfamide-class ligands);
  (b) Zn-O ≤ 2.6 Å for O-donor chemotypes (e.g., carboxylates or phenolates).
  Poses that did not satisfy these criteria were not excluded entirely, but were instead flagged as non-canonical and interpreted either as Zn-proximal alternatives or as rim/entrance-binding hypotheses, depending on their position relative to the catalytic center. These outcomes were therefore not treated as classical Zn-anchored inhibition modes. For transparency, the top-scoring Vina pose (Mode 1) was still reported, but it was not used to infer binding plausibility unless it also fulfilled the Zn-geometry rule.

To provide a direct structural benchmark against the crystallographic sulfonamide reference, we visually compared the selected Tier 1 docking complexes with redocked acetazolamide anion in the CAI active site (Supplementary Figure S1). The comparison supports the geometry-filtered interpretation of the docking results: CID 103935964 (neutral) and CID 112684680 (neutral) both recovered canonical Zn–N-anchored poses with selected Vina scores of −5.122 and −5.799 kcal/mol, respectively, whereas the non-sulfonamide control CID 122367674 (anion) adopted a canonical Zn–O-anchored pose at −5.080 kcal/mol. Redocked acetazolamide anion likewise reproduced a canonical Zn–N interaction (ΔG = −5.18 kcal/mol), providing a useful internal reference for a coordination-credible CAI binding mode. Importantly, the score range across these four complexes is relatively narrow; therefore, the most informative distinction is not score alone, but the recovery of chemically plausible Zn-bound microstates. In this context, the figure visually reinforces the manuscript’s central conclusion that docking in this study is best interpreted as a coordination-plausibility filter, with the two aryl–alkyl sulfamide Tier 1 ligands favoring neutral Zn–N anchoring, while the carboxylate control validates an alternative but still canonical Zn–O binding mode.

### 4.9. Structure–Activity Relationship (SAR) Rationale and Its Role in This Workflow

SAR analysis provides the mechanistic bridge between predicted inhibitory potency and chemically interpretable design rules, and it remains central to rational inhibitor discovery. For CAI (as for other human α-carbonic anhydrases), SAR is particularly sensitive to the identity, accessibility, and protonation state of the zinc-binding group (ZBG), because productive inhibition typically depends on presenting an appropriate donor atom to the catalytic Zn^2+^ [42]. In parallel, potency and isoform bias are often shaped by how polar and lipophilic features are arranged along the scaffold to complement the conical catalytic pocket and its solvent-exposed entrance/rim region, where “tail” substituents can modulate binding mode and off-isoform recognition [43,44]. Even small changes in ZBG substitution, scaffold topology, or polarity distribution can shift binding geometry (canonical Zn-anchored vs. alternative modes), alter potency, and influence cross-isoform liabilities within a highly conserved CA family.

In the present workflow, SAR is not treated as a purely qualitative, post hoc narrative; it is operationalized at three complementary levels. First, descriptor-level attribution (PFI/SHAP) identifies stable chemical drivers underlying the QSAR signal (e.g., sulfur/heteroatom connectivity patterns, polar–lipophilic organization, and scaffold topology), translating statistical prediction into interpretable structural hypotheses. Second, chemotype-level grouping (e.g., N-donor sulfonamide/sulfamide-like scaffolds versus O-donor carboxylates/phenols, and bulky multi-sulfonyl architectures) sets priori expectations for whether a candidate is more likely to support canonical Zn anchoring or adopt rim/entrance-biased binding modes consistent with known CA inhibitor binding diversity. Third, coordination-aware docking serves as a mechanistic plausibility gate by testing whether each QSAR-prioritized chemotype can realize a structurally credible CAI hypothesis under an explicit Zn–donor distance rule, thereby separating canonical Zn-anchored poses from non-canonical Zn-proximal or rim-binding solutions that are often obscured by score-only docking [42].

Practically, this SAR framing enables a tiered and defensible progression strategy: candidates are advanced not because they optimize a single metric, but because they jointly satisfy high predicted potency, coordination-credible binding geometry (or a clearly stated alternative mechanism), and acceptable developability characteristics. In doing so, the workflow reduces false confidence arising from raw docking scores and converts model outputs into testable medicinal chemistry hypotheses that can guide prioritized biochemical validation.

### 4.10. In Silico ADME and Drug-Likeness Profiling

To support early-stage developability triage prior to experimental follow-up, the ten docked CAI candidates were evaluated using the SwissADME web platform [45]. Canonical SMILES were submitted to compute core physicochemical descriptors—molecular weight (MW), topological polar surface area (TPSA), and multiple lipophilicity estimates summarized as consensus logP (o/w)—which jointly inform the solubility–permeability balance relevant to systemic exposure and target engagement. Drug-likeness was assessed using Lipinski’s rule-of-five alongside complementary medicinal chemistry filters implemented in SwissADME, including PAINS and Brenk alerts, to flag substructures associated with assay interference risk or reduced developability.

Absorption and distribution behavior was further profiled using the BOILED-Egg model, which predicts gastrointestinal (GI) absorption and blood–brain barrier (BBB) permeability based on polarity and lipophilicity [46]. This is particularly relevant for systemic CA inhibition, where limiting unintended CNS exposure may be desirable depending on the intended indication. In addition, SwissADME outputs for P-glycoprotein substrate propensity and CYP inhibition flags were recorded to highlight potential transporter- and metabolism-related liabilities that can influence exposure and drug–drug interaction risk. Overall, integrating SwissADME profiling with docking-based structural plausibility provides a practical, decision-oriented filter to prioritize candidates that combine coordination-credible binding hypotheses with manageable developability characteristics, consistent with established in silico triage practice.

## 5. Conclusions, Limitations, and Future Work

### 5.1. Conclusions

This study establishes a reproducible and interpretable QSAR-to-structure triage framework for Carbonic Anhydrase I (CAI) inhibitor discovery. Using ChEMBL CAI records (CHEMBL261), we curated 6958 unique inhibitors and modeled potency on a continuous scale (${pK}_{i}$). A leakage-controlled workflow (train-only fitting of preprocessing and feature selection) reduced 4224 AlvaDesc 2D descriptors to a compact, mechanistically interpretable top-100 panel. Explainability analyses (PFI and SHAP) converged on chemically coherent drivers—sulfur/heteroatom connectivity and polar–lipophilic organization—consistent with established CA inhibitor motifs and scaffold-level complementarity. Among five evaluated regressors, the bagging ensemble provided the strongest test-set generalization ($R^{2}$ = 0.646) and was selected for prospective screening. A PubChem 90% 2D similarity expansion (25,315 analogs) yielded 233 predicted high-potency candidates (${pK}_{i}^{pred}$ ≥ 8.0), of which 145 had independently available PubChem CAI $K_{i}$ values, supporting external transferability under heterogeneous assay provenance ($R^{2}$ = 0.358; RMSE = 0.456; MAE = 0.320).

To translate ligand-based prioritization into structurally testable hypotheses, ten novel candidates lacking CAI $K_{i}$ records were triaged using coordination-aware AutoDock Vina docking (PDB: 1AZM) with explicit Zn–donor geometry filtering. Combined with SwissADME profiling, this evidence enabled a tiered, decision-oriented shortlist that distinguishes high-confidence Zn-anchored candidates from property-limited leads and alternative (rim/entrance) binding hypotheses.

### 5.2. Methodological Limitations

Several constraints should be considered when interpreting the findings:

1. Applicability domain and validation scope: The PubChem expansion and external validation were conducted in near-neighbor chemical space defined by ≥0.90 2D similarity to potent CAI seeds; thus, validation supports transferability within adjacent scaffold space rather than broad “scaffold hopping.”
2. Split strategy and residual correlation: The primary evaluation used an 80/20 split; while leakage was controlled, scaffold-aware splits (e.g., Murcko-based) may provide a stricter estimate of out-of-scaffold generalization. Descriptor blocks can also remain partially correlated after pruning, so interpretability is most defensible at the descriptor-family level.
3. Metalloenzyme docking constraints: AutoDock Vina scoring does not fully parameterize Zn coordination energetics. The Zn–donor distance rule improves plausibility, but docking outputs remain pose hypotheses, not structural proof.
4. Protonation/tautomer sensitivity: Several leads showed protomer dependence for Zn anchoring, emphasizing that automated protonation and charge assignment can affect docking outcomes and should be treated as a controlled experimental factor.
5. In silico developability is predictive: SwissADME outputs are model-based estimates; experimental solubility, permeability, and metabolic stability can differ materially.
6. Isoform selectivity not yet established: The present work prioritizes CAI potency and structural plausibility; selectivity vs. CA II and CA IX/XII remains an essential downstream checkpoint.
7. The QSAR framework relies on 2D molecular descriptors, which do not explicitly encode 3D conformational or protein-context effects. In addition, the docking stage was used as a coordination-aware plausibility filter rather than as a quantitative predictor of binding affinity.

Importantly, the prioritized compounds should be interpreted as computationally supported CAI inhibitor hypotheses rather than experimentally validated leads, and their value now lies in guiding staged biochemical confirmation.

### 5.3. Future Work

To convert these computational hypotheses into validated CAI leads, the next steps should be staged, testable, and explicitly aligned with the selectivity and developability challenges of carbonic anhydrase inhibitor discovery. Because potency alone is not an adequate optimization objective within a highly conserved carbonic anhydrase family, future work must also define isoform selectivity windows and reduce polypharmacology-related risk. The following progression is therefore recommended:

1. Biochemical confirmation with standardized kinetics: Prioritize Tier 1 candidates for CAI inhibition assays under controlled conditions (report $K_{i}$, buffer, pH, temperature, and kinetic model), then expand to Tier 2 and Tier 3.
2. Isoform panel profiling: Quantify cross-reactivity against CA II (off-target) and CA IX/XII (clinically relevant) to define selectivity windows and reduce polypharmacology-related risk.
3. Extension of the workflow beyond CAI: Evaluate whether the present interpretability-guided QSAR-to-structure framework can be extended to related isoforms, particularly CA II and CA IX/XII, to support comparative modeling, selectivity-aware prioritization, and broader translational relevance.
4. Mechanism-of-inhibition testing for Tier 3 hypotheses: For rim/entrance-biased candidates, perform kinetic modality studies (competitive vs. non-competitive/mixed) to determine whether inhibition is Zn-dependent or alternative-site mediated.
5. Structure refinement beyond rigid docking: For the most promising scaffolds, apply metal-aware refinement (e.g., restrained pose optimization and/or MD with appropriate Zn treatment) to evaluate pose stability and reconcile protomer sensitivity.
6. Property-driven optimization: Use the flagged ADME liabilities (e.g., high TPSA, low GI absorption, or CYP alerts) as design constraints for bioisosteric replacement and tail tuning, targeting improved developability while preserving the Zn-binding pharmacophore and SAR-consistent scaffold organization.

## Figures and Tables

**Table 1:** Top 20 AlvaDesc-2D descriptors ranked by bagging importance for CAI $pK_{i}$ prediction.

ID	Descriptor	Scaled Descriptor Importance	% Contribution	Descriptor Family/Chemical Interpretation
1	F03[O–S]	1	0.033	Atom-pair count for O–S pairs at topological distance 3; captures sulfonyl-proximal environments typical of sulfur-rich CA inhibitor chemotypes.
2	SpMax2_Bh(m)	0.574	0.019	Burden eigenvalue descriptor (mass-weighted); reflects overall scaffold size, branching, and mass distribution.
3	CATS2D_04_PL	0.507	0.017	2D pharmacophore descriptor for Positive-Lipophilic pairs at lag 4; reflects polar–lipophilic spacing along the scaffold.
4	SpMax2_Bh(v)	0.463	0.016	Burden eigenvalue descriptor (van der Waals volume-weighted); captures molecular bulk and volume distribution.
5	F02[S–S]	0.296	0.01	Atom-pair count for S–S pairs at topological distance 2; reflects sulfur connectivity patterns often associated with sulfur-containing inhibitor motifs.
6	SpMax2_Bh(e)	0.277	0.009	Burden eigenvalue descriptor (electronegativity-weighted); reflects electronic distribution across the scaffold.
7	arLevel3	0.275	0.009	Aromaticity index; describes aromatic ring complexity and architecture, which may influence scaffold packing and hydrophobic complementarity.
8	B02[S–S]	0.249	0.008	Binary atom-pair descriptor indicating presence of S–S at topological distance 2; a simplified marker of sulfur-rich connectivity.
9	VE1sign_D	0.195	0.007	Distance-matrix eigenvector descriptor; reflects global topology and molecular shape distribution.
10	GATS1s	0.178	0.006	Geary autocorrelation descriptor (intrinsic state-weighted, lag 1); captures short-range distribution of local electronic/structural properties.
11	MATS4s	0.152	0.005	Moran autocorrelation descriptor (intrinsic state-weighted, lag 4); reflects medium-range propagation of local property patterns across the graph.
12	DECC	0.151	0.005	Eccentricity-based descriptor; measures molecular compactness versus extension, relevant to scaffold shape.
13	nArCONR2	0.133	0.004	Functional-group count descriptor; counts aromatic amide-like environments, reflecting heteroatom-rich aromatic substitution patterns.
14	GATS4s	0.13	0.004	Geary autocorrelation descriptor (intrinsic state-weighted, lag 4); captures medium-range organization of structural/electronic properties.
15	SpMax2_Bh(p)	0.125	0.004	Burden eigenvalue descriptor (polarizability-weighted); reflects molecular softness and dispersion-related distribution.
16	P_VSA_charge_13	0.109	0.004	Charge-partitioned van der Waals surface area descriptor; reflects surface charge distribution and possible desolvation/electrostatic effects.
17	MATS2i	0.099	0.003	Moran autocorrelation descriptor (ionization potential-weighted, lag 2); captures reactivity-related property distribution at medium range.
18	VE1_X	0.099	0.003	Adjacency-matrix eigenvector descriptor; reflects graph connectivity and global scaffold organization.
19	MATS1e	0.098	0.003	Moran autocorrelation descriptor (electronegativity-weighted, lag 1); captures local polarity/electronic patterning.
20	SdsCH	0.097	0.003	E-state descriptor for substituted carbon–hydrogen environments; reflects local substitution patterning around carbon centers.

**Table 2:** Training and test performance of candidate regression models.

Model	Key Hyperparameters	Training			Test		
		*R^2^*	RMSE	MAE	*R^2^*	RMSE	MAE
Elastic Net (GLM)	α = 0.5	0.26	0.90	0.71	0.26	0.88	0.70
Regression tree (R tree)	depth = 10; min node = 10	0.54	0.71	0.54	0.29	0.86	0.65
Bagging	500 trees; depth = 50; row = 0.632	0.94	0.25	0.18	0.65	0.61	0.45
Gradient boosting (GB)	100 trees; depth = 10; lr = 0.1	0.96	0.21	0.15	0.64	0.61	0.46
XGBoost (XGB)	100 trees; depth = 10; eta = 0.1	0.98	0.14	0.08	0.64	0.61	0.45

**Table 3:** Selected ten novel molecules prioritized for CAI docking (structural class and rationale). Top-ranked, structurally diverse PubChem candidates without CAI *K_i_* records at the time of retrieval, selected from the QSAR-prioritized subset with $pK_{i}^{pred}\geq 8.0$ for coordination-aware docking against human CAI (PDB: 1AZM). Candidates were chosen to avoid redundant near-isomers and to span mechanistically distinct chemotypes (Zn–N donors, charged sulfonyl scaffolds, and O-donor controls).

ID	PubChem CID	$pK_{i}^{pred}$	PubChem Name	Canonical SMILES	Structural Class/Motif	Rationale for Docking (Coordination-Aware Triage)
1	103935964	9.01	1-methoxy-2-[(1S)-1-(sulfamoylamino)ethyl]benzene	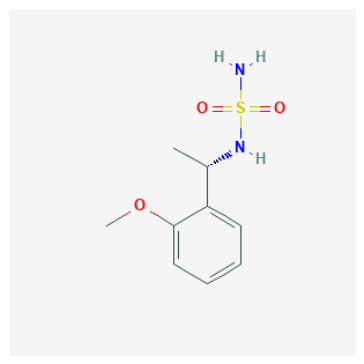	N-alkyl sulfamide (N-donor; o-methoxy aryl–alkyl)	Highest predicted potency; tests whether ortho-methoxy supports a Zn-proximal orientation (direct or water-mediated) while improving entrance packing without compromising sulfamide Zn-anchoring competence.
2	122367674	8.91	trans-(1S,2S)-2-[(2,6-dibromo-3,4-dimethoxyphenyl)methyl]cyclopropane-1-carboxylic acid	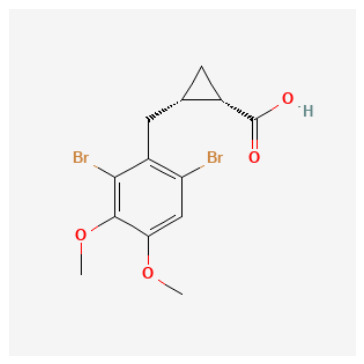	Halogenated aryl–cyclopropyl carboxylic acid (O-donor)	Non-sulfonamide control/extension; probes Zn–O plausibility for a carboxylate and whether halogen-driven shape/electrostatic complementarity can stabilize a coordination-credible pose (QSAR extrapolation stress test).
3	112684680	8.55	2,4-dimethoxy-1-[1-(sulfamoylamino)ethyl]benzene	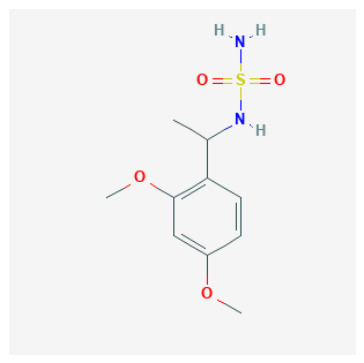	N-alkyl sulfamide (N-donor; dimethoxy aryl–alkyl)	Tests whether dual methoxy substitution improves polar/H-bond acceptor patterning and rim/entrance complementarity while retaining Zn-site plausibility of the sulfamide anchor.
4	101018082	8.46	5-[4-[(7-chloro-2-oxochromen-4-yl)methylamino]phenyl]sulfonylimino-4-methyl-1,3,4-thiadiazole-2-sulfonamide	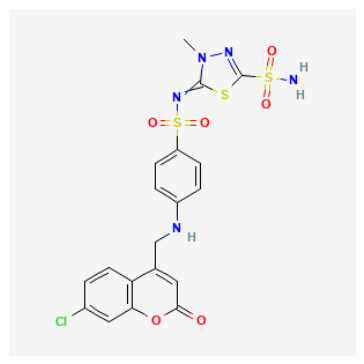	Bulky coumarin–thiadiazole, multi-sulfonyl hybrid (N/O donors)	Multi-point recognition probe; challenges CAI with a bulky scaffold + multiple sulfonyl motifs to test catalytic cone occupancy, rim tolerance, and whether any pose survives Zn-geometry filtering.
5	132523446	8.24	3-(5-hydroxy-1,2,3,4-tetrahydronaphthalen-2-yl)-1,1-dimethylurea	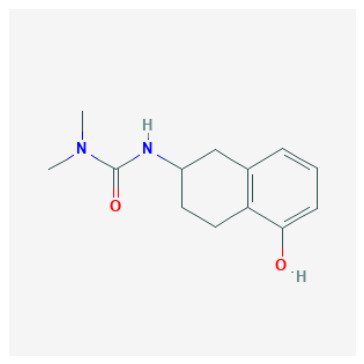	Tetralin–urea/phenol (non-classical; O-donor candidate)	Mechanistic probe; tests whether a hydrophobic fused ring drives entrance/rim binding versus any Zn-proximal O-donor engagement (phenol/phenolate), under the same Zn-centered sampling.
6	118731313	8.3	5-[(4-pyridin-1-ium-1-ylphenyl)sulfonylamino]-1,3,4-thiadiazole-2-sulfonamide	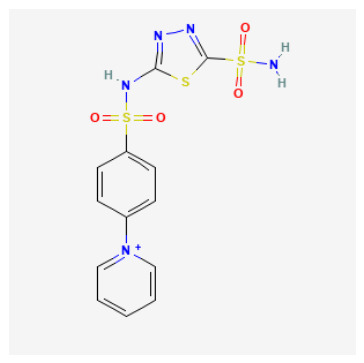	Cationic sulfonamide–thiadiazole (pyridinium; high polarity)	Evaluates electrostatic steering by a permanent cation and whether CAI tolerates high polarity while still allowing a coordination-credible Zn-proximal pose (Zn–N/O filter).
7	72793783	8.3	benzyl N-[(6,7-dimethoxy-1,2,3,4-tetrahydronaphthalen-2-yl)sulfamoyl]carbamate	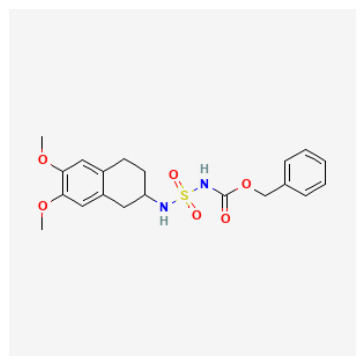	Tail-extended sulfamide (carbamate-linked benzyl tail)	Maps tail-pocket tolerance: flexible carbamate–benzyl extension samples channel/entrance while the sulfamide attempts Zn engagement; useful for distinguishing rim-biased vs. Zn-anchored behavior under geometry filtering.
8	74984350	8.41	[4-[(3-methyl-5-sulfamoyl-1,3,4-thiadiazol-2-ylidene)amino]sulfonylanilino] morpholine-4-carbodithioate	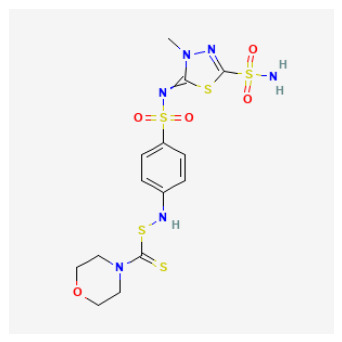	Sulfonamide/sulfamide-rich thiadiazole with thiocarbamoyl–morpholine tail	Tests multi-contact binding: Zn-site engagement from sulfonyl functionality plus a thiocarbamoyl/morpholine tail for rim solvation/H-bonding; also flags potential polarity/CYP/DDI liabilities early during triage.
9	58923319	8.19	1-[(2R)-2-(dimethylsulfamoylamino)propyl]-4-methoxybenzene	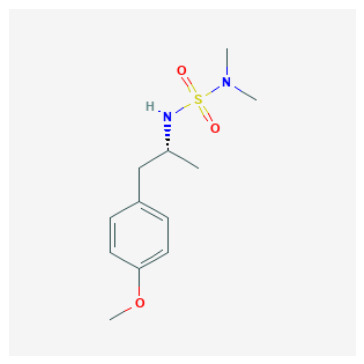	Tertiary sulfamide (N,N-dialkylated; protomer-sensitive)	Probes the SAR boundary of N-alkylation: tertiary substitution may disrupt classical Zn–N anchoring; docking tests whether any Zn-proximal geometry is retained or whether rim/off-Zn modes dominate under filtering.
10	136048763	8.14	5-(4-aminobenzenesulfonylamino)-4,5-dihydro-[1,3,4]thiadiazole-2-sulfonic acid amide	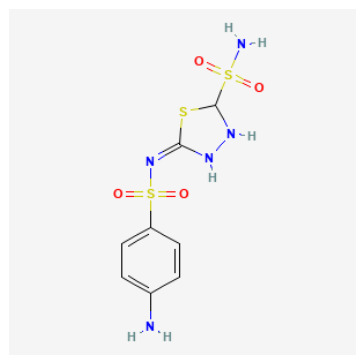	Dual-sulfonyl thioheterocycle (very high polarity)	Tests whether CAI can accommodate a compact but highly polar dual-sulfonyl motif; useful for discriminating true Zn-anchored binding from potential false-positive risk in polar scaffolds.

**Table 4:** Integrated SAR summary for the ten docked leads (QSAR + coordination-aware docking + triage tier).

#	CID	$pK_{i}^{pred}$	Dominant ZBG Class	Best Coordination Evidence (Table S3)	Protomer Sensitivity	Assigned Binding Mode	Triage Tier *
1	103935964	9.01	N-alkyl sulfamide-like (N-donor)	Zn–N 2.37 Å (neutral); borderline 2.71 Å (anion)	Yes	Conditional Zn–N (neutral-favored)	Tier 1
2	122367674	8.91	Carboxylic acid/carboxylate (O-donor)	Zn–O 2.31–2.40 Å (both forms)	Low	Canonical Zn–O (O-donor)	Tier 1
3	112684680	8.55	N-alkyl sulfamide-like (N-donor)	Zn–N 2.59 Å (neutral); Zn–O-prox (anion)	Yes	Conditional Zn–N (near-threshold)	Tier 1
4	101018082	8.46	Sulfonamide/sulfonyl hybrid (N-donor)	Zn–N 2.39–2.42 Å (both forms)	Low	Canonical Zn–N	Tier 2 (ADME flags)
5	132523446	8.24	Phenol/urea (O-donor candidate)	No Zn–O ≤ 2.6 Å	—	Rim/entrance hypothesis	Tier 3
6	118731313	8.3	Sulfonamide (cationic scaffold; N-donor)	Zn–N 2.53–2.59 Å (both forms)	Low	Canonical Zn–N	Tier 2 (polarity/BBB)
7	72793783	8.3	Tail-extended sulfamide-like	Zn–O-prox only; Zn–N absent; rim-biased even in tight box	Yes	Rim/channel-biased	Tier 3
8	74984350	8.41	Sulfonamide/sulfamide-rich + thiocarbamoyl tail	Zn–N 2.40–2.46 Å (both forms)	Low	Canonical Zn–N	Tier 2 (ADME flags)
9	58923319	8.19	Tertiary sulfamide-like	Zn–N > 3.5 Å; Zn–O-prox only	Yes	Non-canonical/off-Zn	Tier 3
10	136048763	8.14	Dual sulfonyl (N-donor present)	Zn–N 2.31–2.46 Å (both forms)	Low	Canonical Zn–N	Tier 2 (polarity/GI)

* Tier definitions (aligned with Section 3.7): Tier 1 = QSAR-high + canonical/near-canonical Zn anchoring + manageable ADME; Tier 2 = canonical anchoring but developability liabilities (TPSA/GI/CYP/BBB flags); Tier 3 = rim/entrance or non-canonical Zn proximity (mechanistic probes until validated).

## Data Availability

The original contributions presented in this study are included in the article/Supplementary Material. Further inquiries can be directed to the corresponding author.

## Supplementary Materials

The following supporting information can be downloaded at: https://www.mdpi.com/article/10.3390/ph19050778/s1, Table S1 lists the 145 PubChem hits with experimentally reported CAI $K_{i}$ values mapped to human CAI (UniProt P00915), reporting the curated assay-derived potency on a consistent scale (including median/min/max and record counts when multiple CAI $K_{i}$ entries existed per CID, with unit harmonization and ${pK}_{i}$ conversion as defined in Methods). Table S2 lists the remaining 88 QSAR-prioritized PubChem hits with no retrievable CAI $K_{i}$ under the approved inclusion/exclusion rules, serving as the prospective candidate set at the time of curation (i.e., missing/unavailable CAI $K_{i}$ evidence, not a potency value). Table S3 summarizes the coordination-aware AutoDock Vina (SwissDock) docking outcomes for the 10 structurally diverse, non-duplicate leads selected from Table S2, including neutral and relevant anionic/protomeric forms, the Zn-centered search setup (PDB: 1AZM), the selected mode under the Zn–donor geometry rule (Zn–N/O ≤ 2.6 Å), and the best-score mode reported for transparency even when off-Zn. Table S4 reports the SwissADME physicochemical and ADME outputs for the same 10 docked ligands, including drug-likeness rules/alerts (Lipinski, Ghose, Veber, Egan, Muegge), solubility estimates, GI absorption/BBB predictions (BOILED-Egg), P-gp substrate likelihood, CYP inhibition flags, PAINS/Brenk alerts, and synthetic accessibility to support staged experimental prioritization. Figure S1: Comparative visualization of selected CA1 docking complexes for the Tier 1 leads and acetazolamide.
