# Supplementary material for: Interpretable QSAR, External PubChem Validation, and Coordination-Aware Docking Enable Tiered Prioritization of Carbonic Anhydrase I Inhibitors
Source: Pharmaceuticals (Basel). 2026 May 15;19(5):778. doi: 10.3390/ph19050778 (PMC13210430; doi:10.3390/ph19050778)
Supplement: Supplementary file 1 [file pharmaceuticals-19-00778-s001.zip › Figure_S1.pdf]

Supplementaly Figure S1

Supplementary Figure S1. Comparative visualization of selected CA1 docking complexes for the Tier 1 leads and acetazolamide.

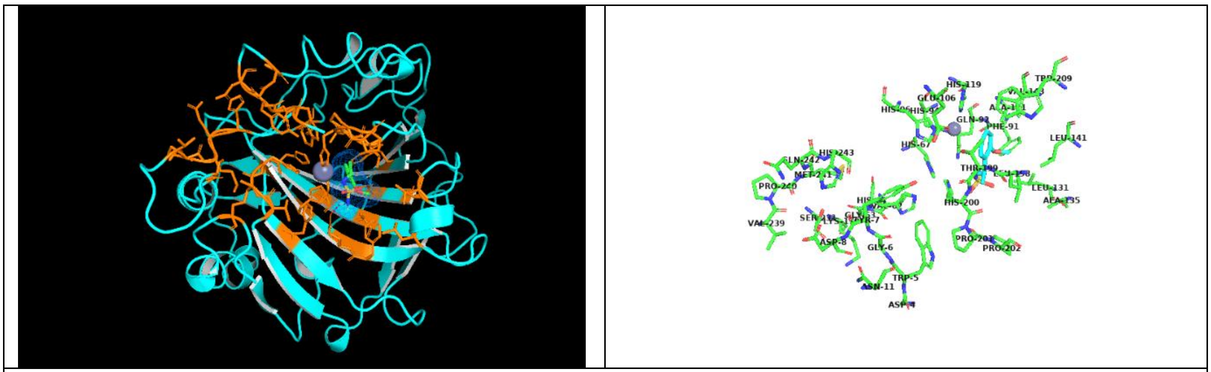

(a) CID 103935964 (neutral)

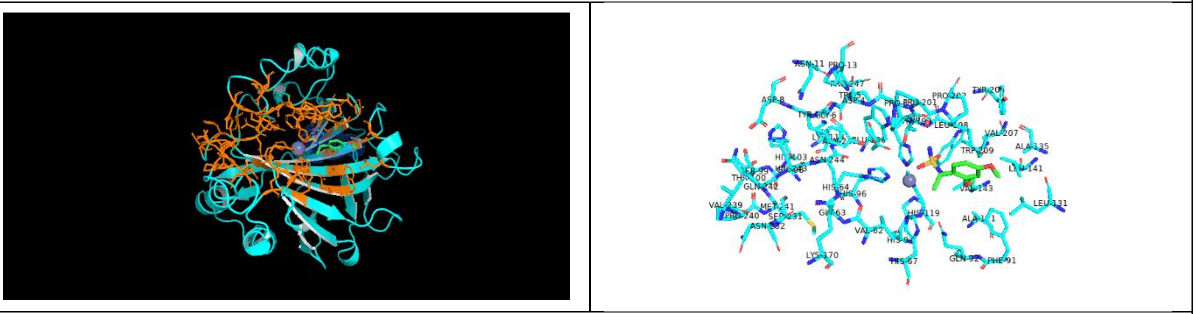

(b) CID 112684680 (neutral)

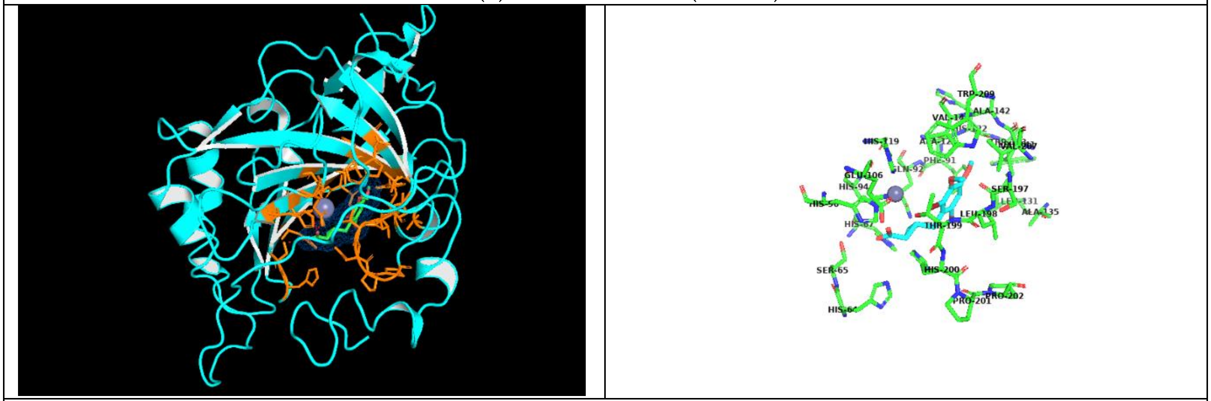

(c) CID 122367674 (anion)

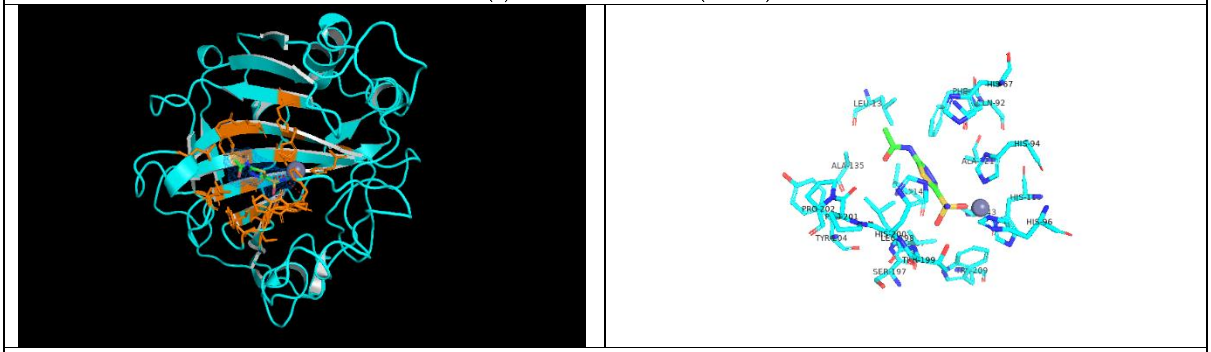

(d) Acetazolamide (anion)

Representative geometry-filtered docking poses in human carbonic anhydrase I (CA1; PDB: 1AZM) are shown for **CID 103935964 (neutral)**, **CID 112684680 (neutral)**, **CID 122367674 (anion)**, and **acetazolamide (anion)**. In each case, the protein is displayed in cartoon representation and the ligand in ball-and-stick form to highlight positioning within the catalytic pocket and proximity to the catalytic Zn^{2+} center. The displayed selected poses

correspond to **CID 103935964: ΔG = −5.122 kcal/mol, Zn–N = 2.37 Å**; **CID 112684680: ΔG = −5.799 kcal/mol, Zn–N = 2.59 Å**; **CID 122367674: ΔG = −5.080 kcal/mol, Zn–O = 2.31 Å**; and **acetazolamide (anion): ΔG = −5.18 kcal/mol, Zn–N = 2.59 Å**. Together, these views illustrate canonical **Zn–N anchoring** for the two sulfamide-like Tier 1 ligands and acetazolamide, and canonical **Zn–O anchoring** for the carboxylate control.
